# *Ab Initio* Polariton Transport Dynamics with the Classical Path Approximation

**DOI:** 10.1021/acs.nanolett.6c00383

**Published:** 2026-04-30

**Authors:** Benjamin X. K. Chng, Braden M. Weight, M. Elious Mondal, Pengfei Huo

**Affiliations:** † Department of Physics and Astronomy, 6927 University of Rochester, Rochester, New York 14627, United States; ‡ Theoretical Division, Center for Integrated Nanotechnologies, 5112 Los Alamos National Laboratory, Los Alamos, New Mexico 87545, United States; ¶ Department of Chemistry, 6927 University of Rochester, Rochester, New York 14627, United States; § The Institute of Optics, Hajim School of Engineering, 6927 University of Rochester, Rochester, New York 14627, United States; ∥ Center for Coherence and Quantum Optics, 6927 University of Rochester, Rochester, New York 14627, United States

**Keywords:** polariton transport, *ab initio* on-the-fly simulations, exciton polariton, light−matter interactions, quantum electrodynamics

## Abstract

We present an *ab initio* framework for simulating polariton transport dynamics based on the classical path approximation (CPA). The quantum dynamics of polariton transport involves simulating many electronic degrees of freedom, making a fully *ab initio* dynamics simulation computationally expensive. We demonstrate that the CPA, which removes the need for excited-state nuclear gradients, is well-suited for polaritonic systems because collective light–matter coupling leads to vanishing excited-state forces. Benchmark comparisons between CPA and full evaluation of the excited-state forces show excellent agreement for polariton transport results in model light–matter systems such as polariton group velocities and mean-squared displacements. *Ab initio* simulations of polariton transport using CPA reproduce key physical trends that are observed in experiments with BODIPY molecules. Our work establishes the CPA as a highly efficient tool for *ab initio* investigations of transport and energy flow in hybrid light–matter systems.

## Graphical abstract

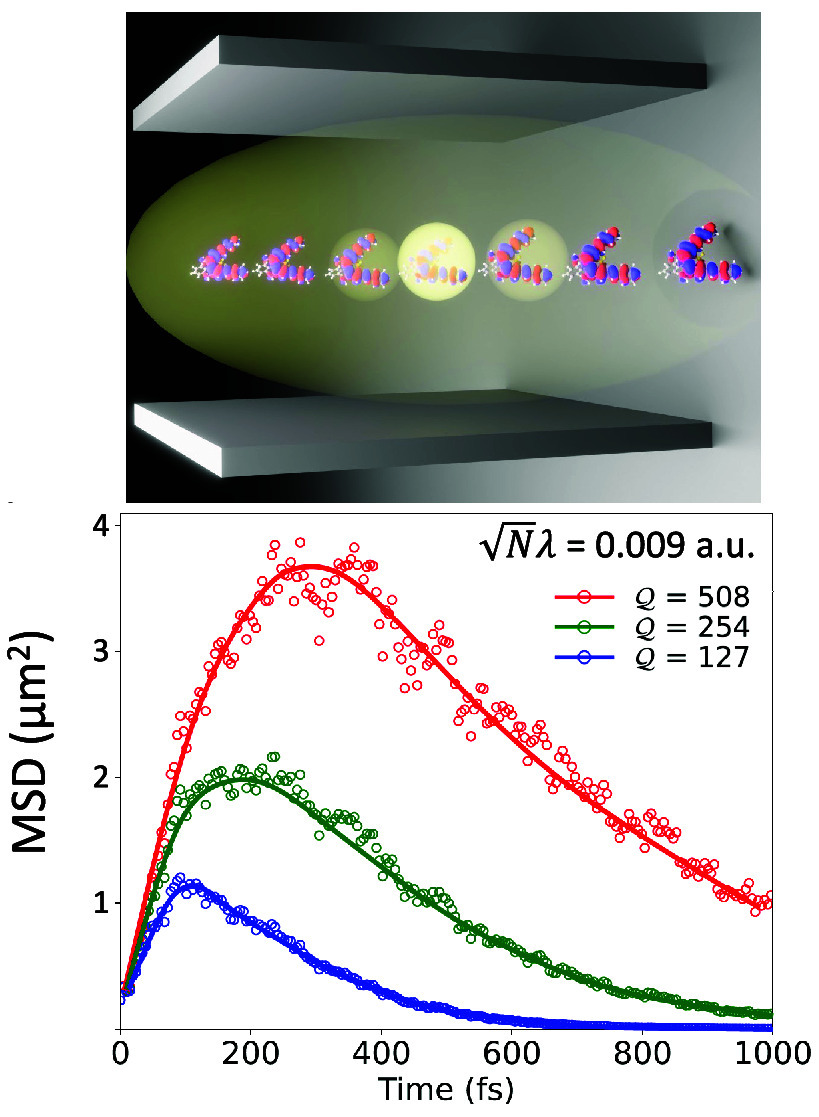

Polaritons, quasi-particles formed by the hybridization of excitons and photons, have recently been shown to enhance energy transport significantly. Recent experiments have reported large group velocities for polaritons.^1–6^ The exciting experimental progress in this area also sparked intensive theoretical investigations^3,7–23^ on polariton transport, using both quantum dynamics simulations^3,19,24^ and analytic theories.^15,17^ Nonetheless, most of these investigations are limited to the simple system-bath type of exciton model systems, with a few exceptions.^9,16^ On-the-fly quantum dynamics simulation is one of the most desirable approaches to explicitly describe the *ab initio* polariton nonadiabatic dynamics in realistic molecule–cavity hybrid systems. Specifically, one requires both ground- and excited-state energies and all state-to-state electric transition and permanent dipole moments, in addition to various response properties of the electronic system such as nuclear gradients of the energies, transition dipole moments, and nonadiabatic couplings,^25^ all at each time step. These evaluations of these gradients make the quantum dynamics simulations in the collective coupling regime computationally prohibitive. In excited-state simulations, a widely used approximation^26–29^ solves the nonadiabatic time-dependent Schrodinger equation (TDSE) on the ground-state classical trajectory. On the other hand, the quantum subsystem would still evolve based on the TDSE for the quantum subsystem. The approximation mentioned above is often termed the classical path approximation (CPA). In this paper, we investigate the validity of the CPA by simulating the quantum dynamics of the generalized Holstein–Tavis–Cummings (HTC) Hamiltonian. The dynamics with CPA show excellent agreement compared to results obtained from the full evaluation of the nonadiabatic and cavity-mediated forces. Further *ab initio* simulations of polariton transport for BODIPY molecules coupled to a FP cavity^2^ are performed in this framework under CPA, and the results reproduce key experimental features, such as the polariton wavepacket’s mean-squared displacement (MSD), from previous experimental work.^2^ The CPA^26–28^ drastically reduces the cost of the electronic structure calculations and enables large-scale, accurate quantum dynamics simulations. In this work, we theoretically demonstrate that the CPA remains valid for the polariton transport dynamics as long as the dynamics themselves are delocalized among many excitonic states.

The molecular Hamiltonian is expressed as

1 $${Ĥ}_{n}={\mathrm{T̂}}_{R_{n}}+E_{g}\left(R_{n}\right)\left|g_{n}\left(R_{n}\right)\right\rangle \left\langle g_{n}\left(R_{n}\right)\right|+E_{e}\left(R_{n}\right)\left|e_{n}\left(R_{n}\right)\right\rangle \left\langle e_{n}\left(R_{n}\right)\right|$$

where ${\mathrm{T̂}}_{R_{n}}$ is the nuclear kinetic energy for molecule *n* and |*g*_*n*_(**R**_*n*_)⟩ and |*e*_*n*_(**R**_*n*_)⟩ are the ground state and excited states, respectively. We use the generalized Tavis–Cummings (GTC) Hamiltonian^3,30–32^ to describe the collective light–matter coupling between molecules and the cavity modes, as follows

2 $$Ĥ=\overset{N}{\underset{n=1}{\sum }}{Ĥ}_{n}+\underset{k_{∥}}{\sum }\hbar \omega_{k}\left({â}_{k}^{†}{â}_{k}+\frac{1}{2}\right)+\underset{k,n}{\sum }\sqrt{\frac{\omega_{k}}{2}}\lambda_{c}{ê}_{k}\cdot {\mathrm{\mu ̂}}_{n}\left(R_{n}\right)\left({â}_{k}e^{ik_{∥}\cdot x_{n}}+{â}_{k}^{†}e^{-ik_{∥}\cdot x_{n}}\right)$$

where ${ê}_{k}$ is the field polarization direction for mode **k**, ${\mathrm{\mu ̂}}_{n}\left(R_{n}\right)$ is the dipole operator of the *n*th molecule, and *x*_*n*_ is the center of mass location of the *n*th molecule.^30^ In addition, λ_c_ is the light–matter coupling strength

3 $$\lambda_{c}=\sqrt{\frac{1}{\epsilon_{0}V}}$$

where V is the cavity effective quantization volume and ϵ_0_ is the permittivity of the materials. In this work, λ_c_ is treated as a parameter.

We model the FP cavity with an open direction *x* characterized by in-plane wavevector **k**_∥_ and one confined direction *z* where **k**_⊥_ is the wavevector of the fundamental mode confined between two cavity mirrors, perpendicular to the mirror surface. The frequency of the cavity mode is given by

4 $$\hbar \omega_{k}=\hbar c\sqrt{{k_{∥}}^{2}+{k_{⊥}}^{2}}$$

where *c* is the speed of light and *ℏω*_c_ = *ℏck*_⊥_ (with *k*_∥_ = 0) is the normal incidence cavity frequency. In addition, ${â}_{k}^{†}$ and ${â}_{k}$ are the photonic creation and annilaition operators for mode **k**, respectively. We consider *k*_∥_ with discrete values $k_{\alpha }=\frac{2\pi }{NL}\alpha$ , where the mode indexes $\alpha \in \left[-\frac{M-1}{2},...0,...\frac{M-1}{2}\right]$ , and M is the total number of cavity modes needed to capture the relevant energies for the hybrid system.

The transport dynamics occur in the single excitation subspace

5a $$\left|E_{n}\left(R\right)\right\rangle =\left|e_{n}\left(R_{n}\right)\right\rangle \underset{m\neq n}{⊗}\left|g_{m}\left(R_{m}\right)\right\rangle \underset{k_{\alpha }}{⊗}\left|0_{k_{\alpha }}\right\rangle$$

5b $$\left|k_{\alpha }\right\rangle =\underset{n}{⊗}\left|g_{n}\left(R\right)\right\rangle \underset{k_{∥}\neq k_{\alpha }}{⊗}\left|0_{k_{∥}}\right\rangle ⊗\left|1_{k_{\alpha }}\right\rangle$$

where |*E*_*n*_⟩ is the singly excited state for the *n*th molecule located at *x*_*n*_ and |*k*_α_⟩ is the one-photon-dressed ground state with photonic momentum *ℏk*_α_.

We use the L -MFE dynamics approach^33–35^ to simulate the polariton transport quantum dynamics in a lossy cavity. This approach combines Meanfield Ehrenfest (MFE) dynamics and Lindblad loss dynamics, describing the exciton-photonic degrees of freedom (DOF) quantum mechanically

6 $$\left|\psi \left(t\right)\right\rangle =\overset{N}{\underset{n=1}{\sum }}c_{n}\left(t\right)\left|E_{n}\left(R\left(t\right)\right)\right\rangle +\underset{\alpha }{\sum }c_{\alpha }\left(t\right)\left|k_{\alpha }\right\rangle$$

The polariton quantum dynamics is propagated with

7 $$i\hbar \frac{\partial }{\partial t}\left|\psi \left(t\right)\right\rangle ={Ĥ}_{Q}\left(R\right)\left|\psi \left(t\right)\right\rangle$$

where ${Ĥ}_{Q}=Ĥ-\underset{n}{\sum }{\mathrm{T̂}}_{R_{n}}\equiv {Ĥ}_{pl}$ is the quantum subsystem Hamiltonian (also the adiabatic polariton Hamiltonian) that includes all DOFs except the nuclear kinetic energy, |ψ­(*t*)⟩ is represented using eq 6, and the EOM is numerically solved by using the RK4 algorithm. Cavity loss is simulated through Lindblad dynamics using a stochastic approach,^33^ assuming identical loss rates Γ_c_ for all cavity modes *k*_α_. We define the cavity quality factor at normal incidence (*k*_∥_ = 0) as

8 $$Q=\hbar \omega_{c}/\Gamma_{c}=\hbar ck_{⊥}/\Gamma_{c}$$

Under the mixed quantum-classical dynamics approximation, the nuclear force is

9 $$F_{n}=-\nabla_{n}E_{g}\left(R_{n}\right)-|c_{n}\left(t\right){|}^{2}\nabla_{n}\left[E_{e}\left(R_{n}\right)-E_{g}\left(R_{n}\right)\right]-\underset{\alpha }{\sum }2Re\left[c_{n}^{*}\left(t\right)c_{\alpha }\left(t\right)e^{-ik_{\alpha }x_{n}}\right]\cdot \nabla_{n}\left\langle g_{n}\right|\lambda_{k}\cdot {\mathrm{\mu ̂}}_{n}\left|e_{n}\right\rangle$$

where ∇_*n*_ ≡ *∂*/*∂* **R**_*n*_ and the excitation population on molecule *n* is |*c*_*n*_(*t*)|^2^ (see eq 6). The second line of eq 9 accounts for the derivative due to the nuclear position dependence of transition dipoles $\lambda_{k}\cdot {\mathrm{\mu ̂}}_{n}\left(R_{n}\right)$ , and we have considered only the transition dipole contributions. In addition, we assume that the center of mass position of molecules is fixed during polariton transport dynamics. This is in agreement with the experimental setup because the molecules are hosted within a PMMA polymer matrix^1,2^ and kept immobilized. The same type of force (eq 9) is also used in mapping-based semiclassical dynamics methods^34–43^ and in the Gaussian wavepacket-based method,^44–46^ and our following discussions on CPA could also be applied to those methods.

Despite the available nuclear gradients and acceleration with the machine learning models,^47^ in general, it is expensive to compute these excited states’ gradients, $\nabla_{R_{n}}E_{e}\left(R_{n}\right)$ . In addition, for the gradient term with transition dipole $\nabla_{n}\left\langle g_{n}\right|\lambda_{k}\cdot {\mathrm{\mu ̂}}_{n}\left(R_{n}\right)\left|e_{n}\right\rangle$ , it is also less straightforward to evaluate, although one can take advantage of the machine learning model.^47^ It is thus ideal to find approximations to avoid explicitly computing these excited states and their dipole-related derivatives.

We hypothesize that under the collective light–matter coupling and in polariton transport dynamics, the CPA is an accurate approximation, such that one does not need to compute the excited states’ gradients as long as polaritons are delocalized among many molecules. This is because throughout the dynamics, the polariton wavepacket is very delocalized, such that most of the |*c*_*n*_|^2^ values are small, with $|c_{n}{|}^{2}∼1/N$ , where N is the number of molecules transiently excited among a total of *N* molecules in the single excitation subspace. Under a truly collective regime for polariton transport dynamics, |*c*_*n*_|^2^ ≪ 1 (for a large N ), and the excited-state force contribution in eq 9 can be ignored. For typical polariton transport experiments, the initial state often has a finite width in *k*_∥_, meaning that the polariton will span a large spatial extent at the initial time, as well as at a later time upon propagation. This is particularly true for the narrow-band excitation used in experiments^1,3^ because the narrower the polariton in *k* space, the more it spreads out in real space. For the broadband excitation used in ref 2, the polariton wavepacket also has a finite speed in space at the initial time (see Figure 2A).

Similarly, the term related to the dipole derivative can be ignored if |*c*_*n*_| ≪ 1, given that |*c*_α_| ≤ 1. This means that we can replace the force in eq 9 with

10 $$F_{n}\approx -\nabla_{n}E_{g}\left(R_{n}\left(t\right)\right)$$

meaning CPA (eq 10) is naturally valid for polariton transport problems. The CPA version of the polariton transport EOM thus uses eq 7 for the quantum subsystem (excitonic and photonic) and eq 10 for the update of the nuclei.

For the sake of simplicity, we have ignored the derivative coupling term ${\mathrm{d̂}}_{n}=\left\langle g_{n}\right|\nabla_{R_{n}}\left|e_{n}\right\rangle$ and Born–Oppenheimer corrections ${\mathrm{D̂}}_{n}=\left\langle g_{n}\right|{\nabla_{R_{n}}}^{2}\left|e_{n}\right\rangle$ in eq 1, which will cause nonradiative relaxation from |*e*_*n*_⟩ to |*g*_*n*_⟩. These processes are less important for the ultrafast polariton transport, as the transport dynamics measured from experiments are usually shorter than the molecular exciton lifetime for the molecules.^1,2^ In addition, the CPA argument can also be applied to the derivative couplings because the forces for those terms scale as $-c_{i}^{*}\left(t\right)c_{j}\left(t\right)\left[d_{ij}\cdot \left(E_{j}-E_{i}\right)\right]$ for electronic states *i* and *j*. Note that **d**_*ij*_ could be large (or even singular) but ∇*V*_*ij*_ = [**d**_*ij*_·(*E*_*j*_ – *E*_*i*_)] is always finite.

We first test the validity of CPA using a system-bath model for the excitonic Hamiltonian (eq 1), allowing us to perform the simulation with the full nuclear gradients, so we can assess the validity of the CPA. The molecular Hamiltonian is

11 $${Ĥ}_{n}=\left(\hbar \omega_{ex}+\lambda_{ex}+\underset{\nu }{\sum }C_{n,\nu }{\mathrm{R̂}}_{n,\nu }\right)⊗{\mathrm{\sigma ̂}}_{n}^{†}{\mathrm{\sigma ̂}}_{n}+\underset{\nu }{\sum }\left(\frac{1}{2}{{\mathrm{P̂}}_{n,\nu }}^{2}+\frac{1}{2}\omega_{\nu }^{2}{{\mathrm{R̂}}_{n,\nu }}^{2}\right)$$

where ${\mathrm{\sigma ̂}}_{n}^{†}=\left|e_{n}\right\rangle \left\langle g_{n}\right|$ , ${\mathrm{\sigma ̂}}_{n}=\left|g_{n}\right\rangle \left\langle e_{n}\right|$ , *ℏω*_ex_ is the exciton energy, and $\lambda_{ex}=\underset{\nu }{\sum }{C_{n,\nu }}^{2}/\omega_{\nu }$ is the reorganization energy, with *C*_*n*,ν_ as the exciton–phonon coupling strength and ω_ν_ as the phonon frequency, and ν is the label for the phonon vibrations. In addition, ${\mathrm{P̂}}_{n,\nu }$ and ${\mathrm{R̂}}_{n,\nu }$ are the momentum and position operators, respectively, of phonon mode ν associated with exciton *n*. Details of the models are provided in the Supporting Information. The light–matter coupling term in eq 2 is replaced with $\sqrt{\omega_{k}/2}\lambda_{k}\cdot {\mathrm{\mu ̂}}_{n}\left(R_{n}\right)\to \hbar g_{c}\sqrt{\frac{\omega_{k}}{\omega_{c}}}\;\cos\theta$ (where tan θ = *k*_∥_/*k*_⊥_), where *g*_c_ is the light–matter coupling strength in the model. Here, we use *N* = 10 001 molecules, M= 141 cavity modes, and reorganization energy λ = 37.2 meV. The simulation results were converged with 250 trajectories using Ehrenfest dynamics, with details provided in the Supporting Information.

Figure 1a presents the impact of varying cavity quality factor Q on *v*_*g*_ with a broadband excitation on the UP band (indicated with the gray Gaussian wavepacket in the inset of Figure 1a), to model experimental conditions in ref 2. The results suggest that group velocity *v*_*g*_ increases with increasing Q , with open circles corresponding to results using the full nuclear force expressions, and the open squares correspond to CPA results, which are in excellent agreement with the full simulation.

**Figure 1:**
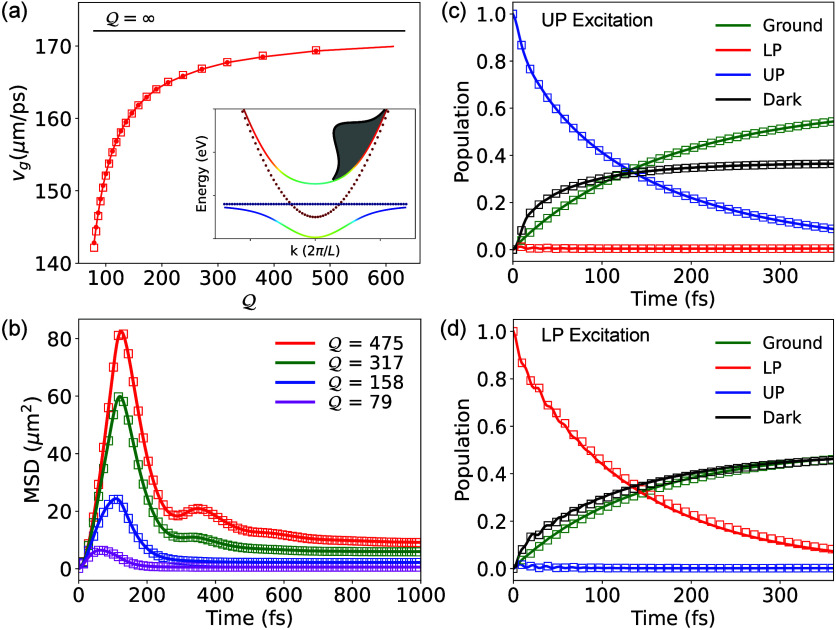
(a) Polariton group velocities *v*_*g*_ vs cavity quality factor Q for wavepackets computed with (filled circles) quantum forces on nuclei and (squares) the CPA. The inset shows the energy bandwidth used for the initial excitation in the UP branch that optimizes the localization of the polariton wavepacket. (b) Time-dependent transient MSD with UP initial excitation for different Q values. The populations of UP (blue), LP (red), dark (black), and ground states (green) in a cavity with Q=475 are presented, with (c) broadband UP excitation and (d) broadband LP excitation.

Figure 1b shows the transient MSD of a polariton wavepacket

12 $$\sigma^{2}\left(t\right)=\left\langle \psi \left(t\right)\right|{\left(\mathrm{x̂}-\left\langle \mathrm{x̂}\right\rangle \right)}^{2}\left|\psi \left(t\right)\right\rangle$$

where ⟨x̂⟩ is the centroid of the initial polariton wavepacket (at *t* = 0) in position space (see the Supporting Information) for details. For cavities with larger Q values, both the wavepacket’s maximum MSD and the corresponding rise time increase. The initial rise of MSD is due to the photonic character of the polariton wavepacket, which is responsible for ballistic transport.^8^ The dip in MSD right after the initial rise is linked to both the decay of the UP population to the dark states and cavity loss, which are competing on a similar time scale.^24^ The empty circles denote MSDs simulated from full calculations, and the empty squares are for MSDs computed with the CPA. As we expect, the MSDs evaluated with the two wavepackets agree well with one another.

Panels c and d of Figure 1 present the population dynamics of the upper polariton (UP) (blue), lower polariton (LP) (red), and dark states (black) under broadband UP excitation and broadband LP excitation, respectively, with cavity quality factors of Q=475 . The ground-state population (green) is also depicted. For *N* molecules and M cavity modes, there are a total of M different UP and LP states each (with different *k*_α_ values) and N−M dark exciton states. The definitions of UP, LP, and dark states are provided in eqs S14 and S15 of the Supporting Information. In panels c and d of Figure 1, the solid lines are populations for wavepackets evaluated with the full nuclear forces while the empty squares are populations evaluated with the CPA. We see that the populations with CPA are in excellent agreement with the full calculations. We have further tested the validity of CPA with various reorganization energies λ and when considering cavity loss dynamics, with results presented in the Supporting Information.

Panels a and b of Figure 2 present the UP polariton wavepacket density and the dark exciton density in the position space for broadband UP excitations. Over time, the UP (blue) wavepacket propagates outward from the center, primarily due to its photonic character, which exhibits ballistic transport (with *v*_*g*_ largely adopted from the derivative of the band). Due to exciton–phonon coupling, the UP wavepackets transfer population to the dark state, resulting in an increase in the dark-state (black) probability densities. The corresponding dark-state wavepacket itself is immobile (due to the zero group velocity of the exciton band), and its spatial expansion is purely due to the expanding UP wavepacket, which deposits populations in the dark exciton states in our simulations. With the full nuclear gradient expression, we can see that the polariton wavepacket remains delocalized among a large number of N sites, leading to a small magnitude of the expansion coefficients of excitons (|*c*_*n*_| ≪ 1) and making the CPA a valid approximation.

**Figure 2:**
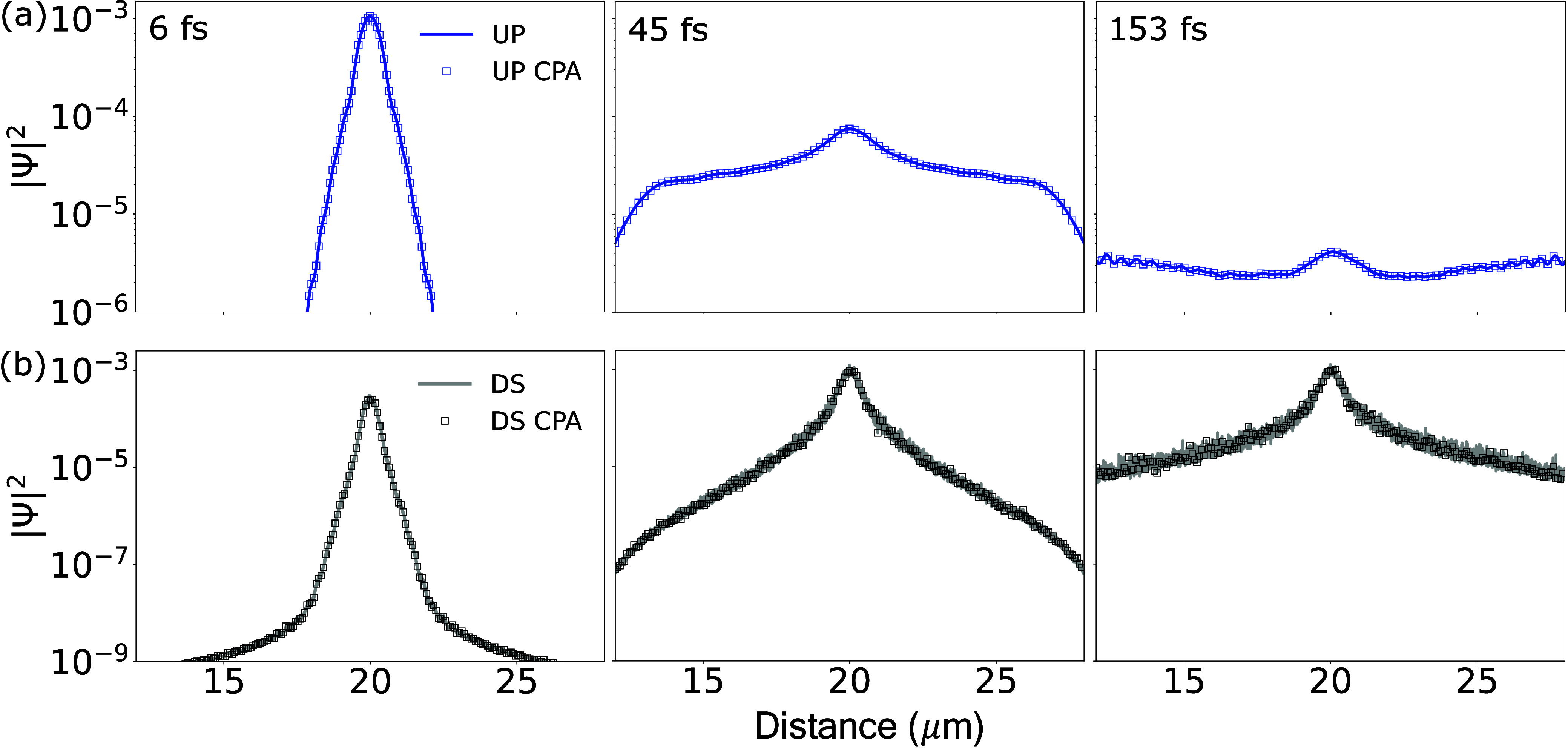
Wavepackets for broadband UP excitation in position space for the (a) UP wavepacket and (b) dark-state wavepacket. The simulations were performed in an FP cavity with Q=475 . The empty squares denote coefficients |*c*_*n*_(*t*)|^2^ evaluated using the CPA.

We also plot the UP wavepacket and the dark-state wavepacket computed with the CPA, and they are denoted in panels a and b, respectively, of Figure 2 with empty squares. The results from CPA match closely with the full simulations. It is clear from Figure 2 that for each molecule at position *x*_*n*_, the coefficients |*c*_*n*_(*t*)|^2^ ≪ 1, satisfying the assumptions needed to use the CPA.

We further use the CPA to simulate *ab initio* polariton transport dynamics for BODIPY coupled to the cavity. The molecular ground-state energies and forces were computed using the semiempirical AM1 Hamiltonian,^48^ while the excited states’ energy and dipoles of these organic molecules were computed using linear-response formalism in the Tamm–Dancoff approximation (TDA-AM1), with our in-house Python code^49^ interfacing with the Gaussian package.^50^ Details are provided in the Supporting Information.

Figure 3 presents *ab initio* simulations of the polariton transmission spectroscopy and transport dynamics of the BODIPY molecules. The experimental investigation of the same system has been done in ref 2. Panels a and b of Figure 3 show the *ab initio* simulation of transmission polariton spectra, with *N* = 108 BODIPY molecules coupled to M=51 FP cavity modes for $\sqrt{N}\lambda$ = 0.01 au and $\sqrt{N}\lambda$ = 0.02 au, respectively. The Rabi splitting is enlarged if one increases the collective light–matter couplings. Here, we present the results as a function of the incident angle, defined as tan θ = *k*_∥_/*k*_⊥_, where *k*_∥_ and *k*_⊥_ are introduced in eq 4. The *k*-resolved (angle-resolved) transmission spectra are computed with

13 $$T_{J}\left(\omega ,k_{\alpha }\right)={\left\langle N_{J,k_{\alpha }}\cdot \delta \left(\hbar \omega -E_{J,k_{\alpha }}\left(R\right)\right)\right\rangle }_{R}$$

where $E_{J,k_{\alpha }}\left(R\right)$ is the polariton energy for the polariton state $\left|\Phi_{J,k_{\alpha }}\right\rangle$ at a given *k*_α_ and $N_{J,k_{\alpha }}=\left\langle \Phi_{J,k_{\alpha }}\left(R\right)\right|{â}_{k}^{†}{â}_{k}\left|\Phi_{J,k_{\alpha }}\left(R\right)\right\rangle$ is the photon number expectation value associated with that polariton state. Computational details for the procedure are provided in the Supporting Information, with a similar computational protocol reported in our earlier work.^51^

**Figure 3:**
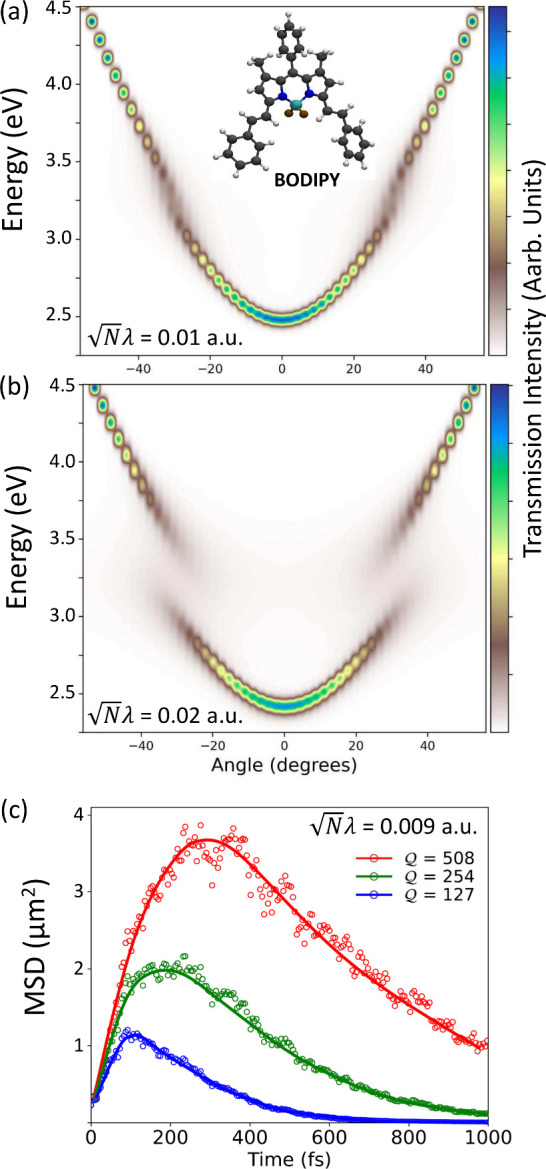
(a and b) Transmission spectroscopy of *N* = 108 BODIPY molecules coupled to M=51 cavity modes at collective light–matter coupling strength (a) $\sqrt{N}\lambda_{c}=0.01$ au and (b) $\sqrt{N}\lambda_{c}=0.02$ au The cavity frequency at *k*_∥_ = 0 is *ℏω*_c_ = 2.50 eV. (c) Mean-squared displacement (MSD) of the polariton wavepacket over time for various cavity quality factors *Q* of (blue, bottom) 127, (green, middle) 254, and (red, top) 508 for initial excitation to the upper polariton. The collective light–matter coupling strength $\sqrt{N}\lambda_{c}\approx 0.009$ au, corresponding to Ω_R_ ≈ 260 meV under the resonant condition between light and matter with the cavity frequency at normal incidence *ℏω*_c_ = 3.0 eV.

Figure 3c shows the *ab initio* simulation results for transient MSD, up to 1 ps, with various Q factors in the CPA framework and for a collective light–matter coupling strength that gives Ω_R_ = 260 meV under the resonance condition. The raw data are presented with empty circles, and the thin curves provide visual guidance. We see that the time-dependent MSDs resemble those measured in the experiments (see Figure 2c of ref 2), validating our on-the-fly simulations of polariton transport dynamics with atomistic and *ab initio* details of the system. Experimentally, the MSD reported in ref 2 is based on the transient absorption measurements. The experimentally measured MSD reported in ref 2 was extracted from the transient differential transmission, and it was better connected through the following expression^8,24^

14 $$\sigma^{2}\left(t\right)=\overset{N}{\underset{n=1}{\sum }}\left(e^{\eta d|\psi \left(x_{n},t\right){|}^{2}}-1\right)\times {\left(x_{n}-\left\langle \mathrm{x̂}\right\rangle \right)}^{2}$$

where η is the sample’s absorption coefficient, *d* is the optical path length, and |ψ­(*x*_*n*_, *t*)|^2^ is the probability distribution function in site basis (see eq S23 of the Supporting Information for details). These two parameters account for the difference between the actual MSD (defined in eq 12) and the experimentally extracted MSD from the differential transmission measurements. The quantity $exp\left(\eta d|\psi \left(x_{n},t\right){|}^{2}\right)-1$ correlates to the transient differential transmission signal Δ*T*/*T* reported in experiments.^2,8^ When *ηd*|ψ­(*x*_*n*_, *t*)|^2^ is small, which is satisfied by the polariton wavepackets, the transient differential transmission signal is approximately $exp\left(\eta d|\psi \left(x_{n},t\right){|}^{2}\right)-1\approx \eta d|\psi \left(x_{n},t\right){|}^{2}$ , and the transient MSD becomes $\sigma^{2}\left(t\right)=\eta d\overset{N}{\underset{n=1}{\sum }}|\psi \left(x_{n},t\right){|}^{2}\times {\left(x_{n}-\left\langle \mathrm{x̂}\right\rangle \right)}^{2}$ ; this expression is identical to the simulated MSD (eq 12) with a scaling factor of *ηd*. We found that *ηd* = 0.0222 reproduces the transient MSD data in ref 2. It is interesting that the *ab initio* simulation MSD behaves qualitatively similar to the HTC model presented in Figure 1b but is quantitatively different. Besides the detailed parameter differences, the realistic *E*_*g*_(**R**_*n*_) and *E*_*e*_(**R**_*n*_) are beyond the simple harmonic phonon bilinearly coupled to exciton as assumed in eq 11, and the dipole vector ${\mathrm{\mu ̂}}_{n}\left(R_{n}\right)$ also fluctuates in time, going beyond a simple constant value. These *ab initio* atomistic features could affect the polariton dynamics. One could use machine learning approaches to build models^47^ to describe these quantities and capture the effects beyond a simple model, but it takes a considerable amount of nontrivial effort.^47^ CPA *ab initio* simulation, as proposed here, avoids these efforts and directly captures how *E*_*g*_(**R**_*n*_), *E*_*e*_(**R**_*n*_), and ${\mathrm{\mu ̂}}_{n}\left(R_{n}\right)$ influence transport dynamics through eqs 2 and 6.

Figure 4 shows the transient MSD of the polariton wavepacket, up to 1 ps, with various Q factors. We show λ_c_ = 0.0017 au, λ_c_ = 0.0070 au, and λ_c_ = 0.014 au in panels a–c, respectively, of Figure 4, corresponding to Ω_R_ = 50 meV, Ω_R_ = 200 meV, and Ω_R_ = 400 meV. The black circles in panels b and c of Figure 4 are the experimental data for ref 2 for *n* = 6.5 layers in the FP cavity. As cavity loss rate Γ_c_ increases, the duration of the ballistic phase, characterized by a sharp increase in the MSD, decreases, in line with previous simulations and experimental measurements. The corresponding peak MSD also decreases as Γ_c_ increases. In addition, we see that experimental results are in close agreement with the *ab initio* simulations for a collective light–matter coupling strength Ω_R_ between 200 and 400 meV and a cavity loss rate Γ_c_ = 10 meV (see panels b and c of Figure 4).

**Figure 4:**
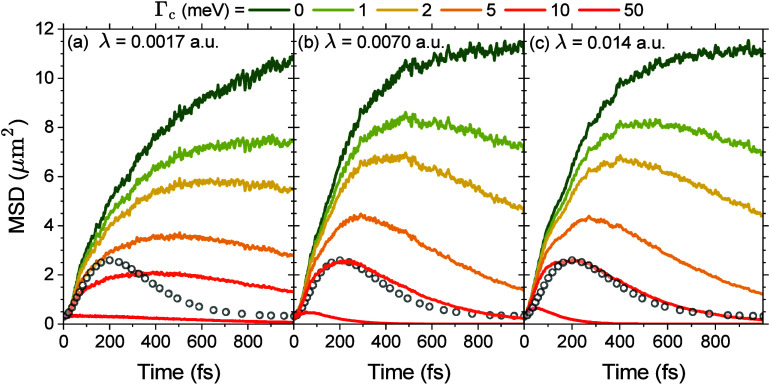
Mean-squared displacement (MSD) of the polariton wavepacket over time for various cavity quality factors Γ_c_ at collective light–matter coupling strengths (a) Ω_R_ = 50 meV (λ_c_ = 0.0017 au), (b) Ω_R_ = 200 meV (λ_c_ = 0.0070 au), and (c) Ω_R_ = 400 meV (λ_c_ = 0.014 au). Black circles are experimental data (scaled) for *n* = 6.5 layers in the distributed Bragg reflector (DBR) cavity in ref 2.

For more general cases in polariton photochemistry or photophysics, the condition |*c*_*n*_(*t*)|^2^ ≪ 1 may not always be fulfilled, especially for polariton photochemistry. In polariton photochemistry,^52^ it was found that the polariton state quickly localizes onto one (or a few molecules), where |*c*_*n*_|^2^ is large, and a local bond-breaking process will happen on that molecule, which does sensitively depend on the excited-state force. For polariton transport, from our results, we found that throughout all *t*, |*c*_*n*_|^2^ ≪ 1 for transport dynamics. In other words, transport dynamics could exhibit localization (due to various types of disorder). For example, when considering static exciton energy disorders, dark states are typically more localized,^11,53–55^ and loss to those dark states could reintroduce localization and non-negligible excited-state force contributions. For these situations, we can instead keep tracking each |*c*_*n*_(*t*)|^2^ in time and start the excited-state gradient calculation only when |*c*_*n*_(*t*)|^2^ is larger than a certain threshold (e.g., when the force contribution is in the range of 1–5% of the ground-state force) and still keep the CPA for all other *n* ≠ *m*. This will still drastically save a great deal of computational cost due to avoiding expensive excited-state gradients and derivative couplings. For a more detailed discussion of the CPA and the potential breakdown of it, see the Supporting Information.

In this work, we demonstrate the validity of the CPA in polariton quantum dynamics simulations. The fundamental reason why CPA works so well for polariton transport is that the polariton wavepacket remains delocalized across a large number of molecules during the dynamics, making individual expansion coefficients |*c*_*n*_(*t*)| ≪ 1. Thus, the excited-state contribution to the nuclear force terms, which is proportional to |*c*_*n*_|^2^, will be negligibly small compared with the ground-state forces. Neglecting the excited-state contributions in on-the-fly simulations for polariton systems in the collective coupling regime is therefore a reasonable approximation. We have tested this approximation with HTC model systems, and the results of CPA are shown to be in excellent agreement with the dynamics with full nuclear gradients. We further use CPA to perform *ab initio* polariton transport simulations with BODIPY molecules coupled to a cavity, with transient MSD results that agree well with experiments.^2^ This work paves the way toward using fully *ab initio* simulations to investigate polariton transport properties in experimentally relevant systems.

## Supplementary Material



## Notes

Details of the model Hamiltonian, polariton quantum dynamics propagation method for model systems, details of the simulations of the *ab initio* polariton transmission spectra, details of the *ab initio* polariton quantum dynamics simulations, additional numerical results, and further discussions of the CPA (PDF)
