# Supplementary material for: Ab Initio Polariton Transport Dynamics with the Classical Path Approximation
Source: Nano Lett. 2026 Apr 30;26(18):6055–62. doi: 10.1021/acs.nanolett.6c00383 (PMC13178133; doi:10.1021/acs.nanolett.6c00383)
Supplement: Supplementary file 1 [file nl6c00383_si_001.pdf]

# Supporting Information: Ab Initio Polariton Transport Dynamics with the Classical Path Approximation

Benjamin X. K. Chng,[†] Braden M. Weight,[‡] M. Elious Mondal,[¶] and Pengfei Huo[*,¶,§,‖]

†*Department of Physics and Astronomy, University of Rochester, Rochester, NY 14627, U.S.A.*

‡*Theoretical Division, Center for Integrated Nanotechnologies, Los Alamos National Laboratory, Los Alamos, NM, 87545, U.S.A.*

¶*Department of Chemistry, University of Rochester, Rochester, NY 14627, U.S.A.*

§*The Institute of Optics, Hajim School of Engineering, University of Rochester, Rochester, NY 14627, U.S.A.*

‖*Center for Coherence and Quantum Optics, University of Rochester, Rochester, New York 14627, U.S.A.*

E-mail: pengfei.huo@rochester.edu

## I. Model Hamiltonian Details

**Generalized Holstein-Tavis-Cummings Hamiltonian.**

We use the generalized Holstein-Tavis-Cummings (GHTC) Hamiltonian[1–3] to describe $N$ molecules collectively coupled to multiple cavity modes as follows

$$\hat{H} = \hat{H}_{\text{ex}} + \hat{H}_{\text{b}} + \hat{H}_{\text{ex-b}} + \hat{H}_{\text{ph}} + \hat{H}_{\text{LM}}, \tag{S1}$$

where $\hat{H}_{\text{ex}}$ is the excitonic Hamiltonian describing $N$ non-interacting molecules, $\hat{H}_{\text{b}}$ is the molecular bath Hamiltonian describing the phonon modes associated with each molecule, $\hat{H}_{\text{ex-b}}$ is the exciton-phonon interaction, $\hat{H}_{\text{ph}}$ is the photonic Hamiltonian describing the electromagnetic modes in a cavity, and $\hat{H}_{\text{LM}}$ is the light-matter interaction term.

The photonic Hamiltonian $\hat{H}_{\text{ph}}$ is expressed as[2–4]

$$\hat{H}_{\text{ph}} = \sum_{\text{k}_{\|}} \hbar\omega_{\mathbf{k}} (\hat{a}^{\dagger}_{\mathbf{k}}\hat{a}_{\mathbf{k}} + \frac{1}{2}), \tag{S2}$$

where $\mathbf{k}$ is the wave vector corresponding to a given cavity mode. Here, we analyze a one-dimensional cavity, with a quasi-continuous open direction $x$ characterized by an in-plane wavevector $k_{\|}$, and a confined direction $z$ where $k_{\perp}$ is the wavevector of the fundamental mode confined between two cavity mirrors, perpendicular to the mirror surface. Consequently, the frequencies of the cavity mode are given by

$$\hbar\omega_{\mathbf{k}} = \hbar c\sqrt{k_{\|}^2 + k_{\perp}^2}, \tag{S3}$$

where $c$ is the speed of the light and we assumed the reflective index inside the cavity is $n_{\text{c}} = 1$. When $k_{\|} = 0$, $\hbar\omega_{\mathbf{k}}(0) = \hbar k_{\perp} = \hbar\omega_{\text{c}}$ which is the typical cavity frequency for a single-mode approximation.

Further, we assume there are $N$ molecules equally spaced a distance $L$ apart (lattice constant) along the $k_{\|}$ direction inside the cavity, and we assume periodic boundary conditions[2] along the $k_{\|}$ direction, such that

$$k_{\alpha} \equiv k_{\|}(\alpha) = \frac{2\pi}{NL}\alpha \tag{S4}$$

where the mode index is $\alpha \in [-\frac{\mathcal{M}}{2}, ...0, ...\frac{\mathcal{M}}{2}]$, and $\mathcal{M}$ is the total number of cavity modes needed to capture the relevant energies for the hybrid system.

The excitonic Hamiltonian $\hat{H}_{\rm ex}$ is given by the expression

$$\hat{H}_{\rm ex} = \sum_{n=0}^{N-1} (\hbar\omega_{\rm ex} + \lambda)\hat{\sigma}_n^\dagger \hat{\sigma}_n, \tag{S5}$$

where $\hbar\omega_{\rm ex} = E_e - E_g$ is the excitation energy between the ground and excited states, $\lambda$ is the reorganization energy due to exciton-phonon coupling, defined in Eq. S12. Furthermore, $\hat{\sigma}_n^\dagger$ and $\hat{\sigma}_n$ correspond to the raising and lowering operators for the excitons (electronic degrees of freedom (DOF)). We consider a linear chain of molecules, each centered at $\mathbf{x}_n$, with a uniform spacing $L = \mathbf{x}_n - \mathbf{x}_{n-1}$, and satisfying the boundary condition $x_N = x_0$, forming a simulation box of size $NL$.

The light-matter interaction $\hat{H}_{\rm LM}$ term is expressed as[1–4]

$$\hat{H}_{\rm LM} = \sum_{\boldsymbol{k},n} \sqrt{\frac{\omega_{\boldsymbol{k}}}{2}} \boldsymbol{\lambda} \cdot \hat{\boldsymbol{\mu}}_n(\mathbf{R}_n)(\hat{a}_{\boldsymbol{k}} e^{i\boldsymbol{k}_\parallel \cdot \mathbf{x}_n} + \hat{a}_{\boldsymbol{k}}^\dagger e^{-i\boldsymbol{k}_\parallel \cdot \mathbf{x}_n}), \tag{S6}$$

where $\mathbf{x}_n = n \cdot L$ is the center of mass location of the $n_{\rm th}$ molecule, $\boldsymbol{\lambda}_{\boldsymbol{k}} = \sqrt{\frac{1}{\epsilon_0 V}} \hat{\mathbf{e}}_{\boldsymbol{k}}$ is the coupling for a given mode and wavevector $\boldsymbol{k}$, $\lambda = |\boldsymbol{\lambda}_{\boldsymbol{k}}|$, and $\hat{\boldsymbol{\mu}}_n(\mathbf{R}_n)$ is the dipole operator of the $n_{\rm th}$ molecule.[1]

In Figs. 1 and 2 in the main text, we consider the configuration of dipoles coupled to the transverse magnetic (TM) polarization (p-polarization), and Eq. S6 simplifies to

$$\hat{H}_{\rm LM} = \sum_{k_\parallel} \sum_{n=0}^{N-1} g_{\mathbf{k}} \left( \hat{a}_{\mathbf{k}}^\dagger \hat{\sigma}_n e^{-ik_\parallel x_n} + \hat{a}_{\mathbf{k}} \hat{\sigma}_n^\dagger e^{ik_\parallel x_n} \right), \tag{S7}$$

where the $k_\parallel$-dependent light-matter coupling strength $g_{\mathbf{k}}$ in Eq. S6 is

$$g_{\mathbf{k}} = \hbar g_{\rm c} \sqrt{\frac{\omega_{\mathbf{k}}(k_\parallel)}{\omega_{\mathbf{k}}(0)}} \cos\theta. \tag{S8}$$

In Eq. S8, $g_{\rm c}$ is the single-molecule coupling strength, $\tan\theta = k_\parallel / k_\perp$, and $\cos\theta = |\hat{\mu} \cdot \hat{\mathbf{e}}|$ account for the relative dipole orientation with respect to the TM field polarization. A schematic illustration can be found in Fig. S1 in Ref. 5.

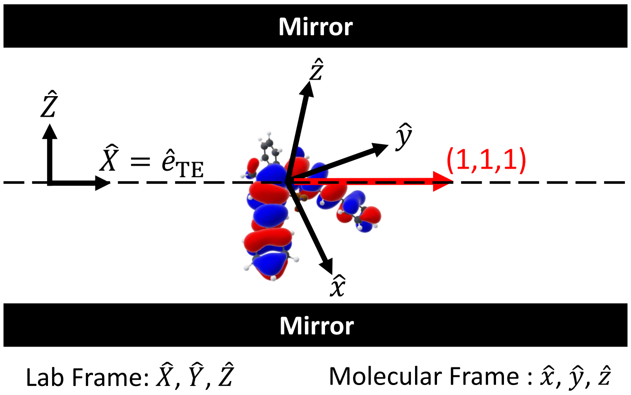


Figure S1: Schematic illustration of a BODIPY molecule inside the optical cavity. In the molecule's reference frame, denoted by $\hat{x}$, $\hat{y}$, and $\hat{z}$ unit vectors, the cavity's electric polarization points along the (1,1,1) direction. In the lab frame (cavity's frame, denoted by $\hat{X}$, $\hat{Y}$, and $\hat{Z}$ unit vectors, the cavity polarization points along the $\hat{X}$ direction. The two frames are related by a set of ordinary 3D-rotations.

For the light-matter coupling term in Eq. S6 in the *ab initio* CPA simulations (reported in Fig. 3c and Fig. 4 of the main text), we define the reference frame of a single BODIPY molecule as residing in the XY-plane. We then choose the TE cavity polarization direction, in the molecular frame, to be $\mathbf{\hat{e}}_{\mathrm{TE}} = (1,1,1)/\sqrt{3}$ for all molecules. In the cavity frame, the TE polarization points $\mathbf{\hat{e}}_{\mathrm{TE}} = \hat{X} = (1,0,0)$. These two frames are related by a set of 3D-rotations.

For the model system, each exciton is coupled to its own phonon bath modes, with the bath Hamiltonian described by

$$\hat{H}_{\mathrm{b}} = \sum_{n=0}^{N-1} \sum_{\nu} \left( \frac{1}{2} \hat{P}_{n,\nu}^2 + \frac{1}{2} \omega_\nu^2 \hat{R}_{n,\nu}^2 \right), \tag{S9}$$

and the system-bath (exciton-phonon) coupling term described by

$$\hat{H}_{\mathrm{sb}} = \sum_{n=0}^{N-1} \hat{\sigma}_n^\dagger \hat{\sigma}_n \otimes \sum_{\nu} C_{n,\nu} \hat{R}_{n,\nu}. \tag{S10}$$

Here, $\omega_\nu$ is the frequency for the $\nu_{\mathrm{th}}$ phonon mode, $\hat{R}_{n,\nu}$ and $\hat{P}_{n,\nu}$ are the position and momentum operators for the $\nu_{\mathrm{th}}$ vibrational mode in the $n_{\mathrm{th}}$ molecule. The bath coupling constants, $c_{n,\nu}$, are

models as the Debye spectral density[6]

$$\mathcal{J}(\omega)=\frac{\pi}{2}\sum_{\nu}\frac{C_{n,\nu}^2}{\omega_{n,\nu}}\delta(\omega-\omega_{n,\nu})=\frac{2\lambda\omega_{\mathrm{b}}\omega}{\omega_{\mathrm{b}}^2+\omega^2}, \tag{S11}$$

where $\lambda$ is the reorganization energy as presented in Eq. S5, and $\omega_{\mathrm{b}}$ is the characteristic frequency of the phonon bath. Further, $\lambda$ can be formally defined as

$$\lambda=\frac{2}{\pi}\int_0^{\infty}d\omega\frac{\mathcal{J}(\omega)}{\omega}=\sum_{\nu}\frac{C_{n,\nu}^2}{\omega_{n,\nu}^2}. \tag{S12}$$

**Polaritonic States and Dark States.**

The polaritonic states are defined from the GHTC Hamiltonian without the bath and exciton-phonon terms. Here, we consider the Hamiltonian $\hat{H}_{\mathrm{pl}}$ as follows

$$\hat{H}_{\mathrm{pl}}=\hat{H}_{\mathrm{ex}}+\hat{H}_{\mathrm{ph}}+\hat{H}_{\mathrm{LM}}, \tag{S13}$$

which excludes the exciton-phonon coupling $\hat{H}_{\mathrm{ex-b}}$ in the polariton Hamiltonian, as opposed to the previous work that includes them in the definition of the polariton Hamiltonian.[3,7]

We define the bright states $|\mathrm{B}_\alpha\rangle$ and dark states $|\mathrm{D}_\kappa\rangle$ of the collective matter exciton as

$$|\mathrm{B}_\alpha\rangle=\frac{1}{\sqrt{N}}\sum_{n=0}^{N-1}\exp\Big(-2\pi i\frac{n\alpha}{N}\Big)|E_n\rangle=\frac{1}{\sqrt{N}}\sum_{n=0}^{N-1}\exp(-ik_\alpha x_n)|E_n\rangle,\quad \alpha\in\{-\frac{\mathcal{M}}{2},\cdots,\frac{\mathcal{M}}{2}\}, \tag{S14a}$$

$$|\mathrm{D}_\kappa\rangle=\frac{1}{\sqrt{N}}\sum_{n=0}^{N-1}\exp\Big(-2\pi i\frac{n\kappa}{N}\Big)|E_n\rangle=\frac{1}{\sqrt{N}}\sum_{n=0}^{N-1}\exp(-ik_\kappa x_n)|E_n\rangle, \tag{S14b}$$

where the $\kappa$ index for the dark states is

$$\kappa\in\left\{-\frac{N}{2},\cdots,-\left(\frac{\mathcal{M}}{2}+1\right)\right\},\ \text{ and }\kappa\in\left\{\frac{\mathcal{M}}{2}+1\cdots,\frac{N}{2}\right\}, \tag{S15}$$

and there are a total of $N-\mathcal{M}$ of dark states. Further, $k_\alpha=2\pi\alpha/(NL)$, $k_\kappa=2\pi\kappa/(NL)$, and $x_n=nL$. The value $\kappa$ does not have a matching $\alpha$ index, and thus, these dark states will not mix with the photonic DOF with $k_\alpha$. Further, we set the indices in Eq. S14b such that the $\alpha_{\mathrm{th}}$ bright state couples to a photon mode with wave-vector $k_\alpha$. Additionally, we define dark states $|\mathrm{D}_\kappa\rangle$ with

indices $\kappa$ that are greater than $\pm\mathcal{M}/2$ (c.f. Eq. S14b).

Due to the light-matter coupling, the bright states $|\mathrm{B}_\alpha\rangle$ hybridize with the photonic states. In the current model, there are a total of $\mathcal{M}$ photonic states $\{|k_\alpha\rangle\}$ and a total of $\mathcal{M}$ bright exciton states (Eq. S14b). The hybridization of bright exciton states and photonic states generates a total of $2\mathcal{M}$ upper and lower polariton states, expressed as

$$|+, k_\alpha\rangle = \cos\Theta_N|\mathrm{B}_\alpha\rangle + \sin\Theta_N|k_\alpha\rangle, \tag{S16a}$$

$$|-, k_\alpha\rangle = -\sin\Theta_N|\mathrm{B}_\alpha\rangle + \cos\Theta_N|k_\alpha\rangle, \tag{S16b}$$

where the mixing angle $\Theta_N$ is

$$\Theta_N = \frac{1}{2}\tan^{-1}\left(\frac{2\sqrt{N}g_{\mathbf{k}}(k_\alpha)}{\omega_{\mathrm{ex}} + \lambda - \omega_{\mathbf{k}}(k_\alpha)}\right) \in [0, \frac{\pi}{2}). \tag{S17}$$

The dark states (Eq. S14) on the other hand, do not couple to the photonic DOF because there is no matching $k$ index from the photonic DOF. These diabatic definitions of polariton and dark states will be used to interpret the quantum dynamics of the population relaxation process presented in Fig. 1 and Fig. 2 of the main text.

**GHTC Model Parameters.**

In simulations of the GHTC model Hamiltonian, the excitonic energy was taken to be $\hbar\omega_{\mathrm{ex}} =$ 1.96 eV, and the fundamental photon frequency was taken to be $\hbar\omega_{\mathrm{c}} = 1.90$ eV (which is $\omega_{\mathbf{k}}$ at the normal incidence $k_\parallel = 0$) for the model used in Figs. 1-2 in the main text. There, $N = 10001$ molecules and $\mathcal{M} = 283$ modes were chosen, keeping the ratio of $N/\mathcal{M} \approx 35$. The inter-molecular spacing is set to be $L = 40$ Å, and the total light-matter coupling strength is fixed at $\sqrt{N}g_{\mathrm{c}} = 120$ meV.

**Spacial Distribution of the Polariton Wavepacket**. To interpret the spatial distribution of the polariton wavepacket at time $t$ and position $x_n$, we define the time-dependent polariton state (without the ground state component)

$$|\psi(t)\rangle = \sum_{n=0}^{N-1} c_n(t)\,|E_n\rangle + \sum_\alpha c_\alpha(t)\,|k_\alpha\rangle \equiv |\psi_{\mathrm{ex}}(t)\rangle + |\psi_{\mathrm{ph}}(t)\rangle, \tag{S18}$$

and define the spatial distribution due to the excitonic part $|\psi_{\rm ex}(t)\rangle$ and photonic part $|\psi_{\rm ph}(t)\rangle$ separately. The polariton states $|\pm, n\rangle$ in real space are obtained by taking a discrete Fourier transform of Eqs. S16a and S16b using the basis $e^{ik_\alpha x_n} = e^{i\frac{2\pi\alpha}{NL}\cdot(nL)} = e^{i\frac{2\pi n\alpha}{N}}$.

This leads to the following expressions

$$|+, n\rangle = \sum_\alpha \left[\sum_{n'=0}^{N-1} \frac{X_{k_\alpha}}{N} e^{ik_\alpha(x_n - x_{n'})}|E_{n'}\rangle + C_{k_\alpha}\frac{e^{ik_\alpha x_n}}{\sqrt{N}}|k_\alpha\rangle\right], \tag{S19a}$$

$$|-, n\rangle = -\sum_\alpha \left[\sum_{n'=0}^{N-1} \frac{C_{k_\alpha}}{N} e^{ik_\alpha(x_n - x_{n'})}|E_{n'}\rangle - X_{k_\alpha}\frac{e^{ik_\alpha x_n}}{\sqrt{N}}|k_\alpha\rangle\right], \tag{S19b}$$

where $C_{k_\alpha} = \sin\Theta_N$ and $X_{k_\alpha} = \cos\Theta_N$ are the Hopfield coefficients of the polariton state with wavevector $k_\alpha$. The spatial distribution of the polariton wavepacket is thus given by $|\psi_\pm(x_n, t)|^2 = |\langle\pm, n|\psi(t)\rangle|^2$, and the full expression for $|\psi_\pm(x_n, t)|^2$ is

$$\begin{aligned}
|\psi_+(x_n, t)|^2 = &\left|\sum_\alpha \sum_{n'=0}^{N-1} \frac{X_{k_\alpha}}{N} e^{ik_\alpha(x_n - x_{n'})} c_n'\right|^2 + \left|\sum_\alpha C_{k_\alpha}\frac{e^{ik_\alpha x_n}}{\sqrt{N}} c_\alpha\right|^2 \\
&+ 2\mathrm{Re}\left[\sum_\alpha \sum_{n'=0}^{N-1} \frac{X_{k_\alpha}}{N} e^{ik_\alpha(x_n - x_{n'})} c_n' \times \left(\sum_{\alpha'} C_{k_{\alpha'}}\frac{e^{ik_{\alpha'} x_n}}{\sqrt{N}} c_{\alpha'}\right)^*\right], \qquad \text{(S20a)} \\
|\psi_-(x_n, t)|^2 = &\left|\sum_\alpha \sum_{n'=0}^{N-1} \frac{C_{k_\alpha}}{N} e^{ik_\alpha(x_n - x_{n'})} c_n'\right|^2 + \left|X_{k_\alpha}\frac{e^{ik_\alpha x_n}}{\sqrt{N}} c_\alpha\right|^2 \\
&- 2\mathrm{Re}\left[\sum_\alpha \sum_{n'=0}^{N-1} \frac{C_{k_\alpha}}{N} e^{ik_\alpha(x_n - x_{n'})} c_n' \times \left(\sum_{\alpha'} X_{k_{\alpha'}}\frac{e^{ik_{\alpha'} x_n}}{\sqrt{N}} c_{\alpha'}\right)^*\right]. \qquad \text{(S20b)}
\end{aligned}$$

The last term in Eq. S20a and Eq. S20b describes the interference between the excitonic part and the photonic part of the polariton wavepacket.

The dark state wavepacket in real space is obtained by taking the discrete Fourier transform of Eq. S14, and this gives

$$|\mathrm{D}, n\rangle = \sum_\kappa \frac{1}{N}\sum_{n'=0}^{N-1} e^{ik_\kappa(x_n - x_{n'})}|E_{n'}\rangle. \tag{S21}$$

The spatial distribution of the dark state wavepacket is given by $|\psi_{\rm D}(x_n, t)|^2 = |\langle\mathrm{D}, n|\psi(t)\rangle|^2$, and is computed as

$$|\psi_{\rm D}(x_n, t)|^2 = \left|\sum_\kappa \frac{1}{N}\sum_{n'=0}^{N-1} e^{ik_\kappa(x_n - x_{n'})} c_{n'}\right|^2. \tag{S22}$$

The above polariton and dark state density dsitribuitons are used in Fig. 2 of the main text.

**Transient MSD simulations.** We compute the polariton group velocities and MSDs from the contributions of the polariton and dark state wavepackets

$$|\psi(x_n,t)|^2 = |\psi_+(x_n,t)|^2 + |\psi_{\mathrm{D}}(x_n,t)|^2, \tag{S23}$$

where we only count the $|+\rangle$ and dark state contribution, for the MSD reported in Fig. 1 of the main text.

The transient MSD is expressed as

$$\sigma^2(t) = \langle\psi(t)|(\hat{x}-\langle\hat{x}\rangle)^2|\psi(t)\rangle = \sum_n |\psi(x_n,t)|^2 \cdot (x_n - \langle\hat{x}\rangle)^2, \tag{S24}$$

where $\langle\hat{x}\rangle$ is the centroid of the initial polariton wavepacket (at $t = 0$) in position space. We use this expression to compute the transient MSD for the model systems (Fig. 1b in the main text).

## II. Polariton Quantum Dynamics Propagation Method For Model Systems

In this work, we treat the excitonic and photonic systems as the quantum DOF, while the phonon bath as the classical DOF. By doing this, we describe the polariton dynamics associated with $\hat{H}_{\mathrm{Q}} = \hat{H} - \hat{H}_{\mathrm{b}}$ using TDSE and evolve the phonon bath DOF associated with $\hat{H}_{\mathrm{b}} + \hat{H}_{\mathrm{ex-b}}$ using Ehrenfest mean-field force. Here, we consider the single excitation subspace in our simulation. In this subspace, we consider the following matter or photonic excitations

$$|E_n\rangle = |\mathrm{e}_n\rangle \bigotimes_{m\neq n} |\mathrm{g}_m\rangle \bigotimes_{k_\parallel\in\{k_\alpha\}} |0_{k_\parallel}\rangle \tag{S25a}$$

$$|k_\alpha\rangle = |G\rangle \bigotimes_{k_\parallel\neq k_\alpha} |0_{k_\parallel}\rangle \otimes |1_{k_\alpha}\rangle\,, \tag{S25b}$$

where $|\mathrm{g}_n\rangle$ and $|\mathrm{e}_n\rangle$ represent the ground and excited states of the $n_{\mathrm{th}}$ molecule, respectively, and $|G\rangle = \bigotimes_n |g_n\rangle$ represents the matter ground state. The excitation of the $\alpha_{\mathrm{th}}$ photon mode with in-plane wave-vector $k_\alpha$ is given by $|1_{k_\alpha}\rangle$, while $|0_{k_\alpha}\rangle$ represents the vacuum state of the $\alpha_{\mathrm{th}}$ photonic mode.

**Details of the $\mathcal{L}$-MFE approach.**

To perform the transport simulations, Ehrenfest dynamics is employed, where we simulate an ensemble of trajectories with varied initial conditions. Within each trajectory, the exciton-photon wavefunction is propagated in accordance with the equation

$$-i\hbar\dot{\mathbf{c}}(t) = \hat{H}_{\mathrm{Q}}(\mathbf{R}(t))\mathbf{c}(t), \tag{S26}$$

where $\mathbf{c}(t) = [\{c_n(t)\}, \{c_\alpha(t)\}]^{\mathrm{T}}$ are the expansion coefficients, with a total of $N$ exciton coefficients $c_n(t)$, a total of $\mathcal{M}$ photonic coefficient $c_\alpha(t)$. The nuclear coordinates are updated according to Newton's equation of motion

$$\frac{\partial R_{n,\nu}}{\partial t} = P_{n,\nu}, \quad \frac{\partial P_{n,\nu}}{\partial t} = \mathcal{F}_{n,\nu} \tag{S27}$$

where the bath modes experience the force from the potential energy surface generated by $|\psi(t)\rangle$,

$$\begin{aligned}\mathcal{F}_{n,\nu} &= -\nabla_{R_{n,\nu}}\langle\psi(t)|(\hat{H}_{\mathrm{ex-b}} + \hat{H}_{\mathrm{b}})|\psi(t)\rangle \\ &= -\omega_\nu^2 R_{n,\nu} - |c_n(t)|^2 \cdot C_{n,\nu},\end{aligned} \tag{S28}$$

where we used the diagonal structure of bi-linear system-bath interaction in the HTC Hamiltonian (Eq. S10), and only $|E_n\rangle$ has the exciton-phonon coupling (whereas $|k_\alpha\rangle$ does not). Here, $C_{n,\nu}$ is the exciton-phonon coupling strength between phonon mode $R_{n,\nu}$ and exciton state $|E_n\rangle$, see Eq. S10. The CPA approximation corresponds to explicitly taking the $|c_n(t)|^2 \to 0$ limit, and resulting in $\mathcal{F}_{n,\nu} \approx -\omega_\nu^2 R_{n,\nu}$ for the HTC model Hamiltonian.

The cavity loss (photon loss) dynamics from state $|k_\alpha\rangle$ to state $|G\rangle$ is formally described using the Lindblad super operator $\hat{L}_\alpha = |G\rangle\langle k_\alpha|$. The dissipator $\mathcal{L}$ accounts for the cavity loss channel, causing the system to relax

$$\mathcal{L}[\hat{\rho}_{\mathrm{Q}}] = \sum_\alpha \Gamma_{\mathrm{c}}\Big(\hat{L}_\alpha\hat{\rho}_{\mathrm{Q}}\hat{L}_\alpha^\dagger - \frac{1}{2}\{\hat{L}_\alpha^\dagger\hat{L}_\alpha, \hat{\rho}_{\mathrm{Q}}\}\Big), \tag{S29}$$

which can be formally unravelled[8] as

$$c_\alpha(t + dt) = c_\alpha(t)e^{-\Gamma_{\mathrm{c}}dt}, \tag{S30}$$

and the coefficients of the ground state change by the amount

$$c_0(t+dt) = e^{i\phi}\sqrt{|c_0(t)|^2 + (1-e^{-\Gamma_\alpha dt})|c_\alpha(t)|^2}, \tag{S31}$$

where the random phase $\phi$ is drawn from a uniform distribution

$$\mathcal{P}(\phi) = \frac{1}{2\Delta_\phi} \quad , \quad \Delta_\phi \le \phi \le \Delta_\phi \tag{S32}$$

and the width of this distribution is determined by numerically solving the transcendental equation

$$\frac{\sin(\Delta_\phi)}{\Delta_\phi} = \frac{|c_0(t)|}{\sqrt{|c_0(t)|^2 + (1-e^{\Gamma_c dt})|c_\alpha(t)|^2}}. \tag{S33}$$

Details of this algorithm can be found in Ref. 8.

## III. Details of Quantum Dynamics Simulations in the Model Systems.

**Polaritonic Initial Conditions**.

We used polariton wavepackets that are localized over the molecules but centered around a given value of $k_\alpha$ as the initial conditions. To generate these wavepackets, we define the polariton states $|\Phi_J\rangle$ as follows

$$\hat{H}_{\text{pl}}|\Phi_J\rangle = \mathcal{E}_J|\Phi_J\rangle, \tag{S34}$$

where $\hat{H}_{\text{pl}} = \hat{H}_{\text{ex}} + \hat{H}_{\text{ph}} + \hat{H}_{\text{LM}}$, and the state $|\Phi_J\rangle$ has the polariton energy $\mathcal{E}_J$. Additionally, we note that the polariton states obtained from diagonalization take the form

$$|\Phi_J\rangle = \sum_n \tilde{c}_{n,J}|E_n\rangle + \sum_\alpha \tilde{c}_{\alpha,J}|k_\alpha\rangle, \tag{S35}$$

where $\tilde{c}_{n,J}$ and $\tilde{c}_{\alpha,J}$ are the excitonic and photonic components of the $J_{\text{th}}$ polariton state, respectively.

To obtain a spatially localized polaritonic initial state, corresponding to a specific energy window $\mathcal{E} \in [\mathcal{E}_0 - \Delta\mathcal{E}/2, \mathcal{E} + \Delta\mathcal{E}/2]$ (generated from initial laser excitation), we expand the initial state in

terms of the polariton states defined in Eq. (S34) as follows

$$|\psi(0)\rangle = \sum_J a_J |\Phi_J\rangle, \tag{S36}$$

where $a_J = \langle \Phi_J | \psi(0) \rangle$ is the expansion coefficient of the initial state $|\psi(0)\rangle$ onto $|\Phi_J\rangle$, with $a_J$ yet to be determined. For the initial excitation in the energy window $\mathcal{E} \in [\mathcal{E}_0 - \Delta\mathcal{E}/2, \mathcal{E} + \Delta\mathcal{E}/2]$, we consider those $\{|\Phi_J\rangle\}$ such that the polariton energy $\mathcal{E}_J \in [\mathcal{E}_0 - \Delta\mathcal{E}/2, \mathcal{E} + \Delta\mathcal{E}/2]$. The coefficients $a_J$ are then generated by minimizing the spread of the wave packet $\Delta x^2$ in the excitonic space, defined as[3]

$$\Delta x^2 = \sum_n \rho_n x_n^2 - \sum_n (\rho_n x_n)^2, \tag{S37}$$

where $\rho_n = |\langle E_n | \psi(0) \rangle|^2 / \sum_m |\langle E_m | \psi(0) \rangle|^2$ is the normalized probability of occupying molecule located at $x_n$, and $|E_n\rangle$ is defined in Eq. S25a. In the single-excited diabatic basis, $\rho_n$ is expressed as

$$\rho_n = \frac{|\sum_J a_J \tilde{c}_{n,J}|^2}{\sum_m |\sum_J a_J \tilde{c}_{m,J}|^2}, \tag{S38}$$

and the spread of the wavepacket $\Delta x^2$ is given by

$$\Delta x^2 = \sum_n \frac{x_n^2 |\sum_J a_J \tilde{c}_{n,J}|^2}{\sum_m |\sum_J a_J \tilde{c}_{m,J}|^2} - \left( \frac{x_n |\sum_J a_J \tilde{c}_{n,J}|^2}{\sum_m |\sum_J a_J \tilde{c}_{m,J}|^2} \right)^2. \tag{S39}$$

Eq. (S39) is used as input in the basin hopping algorithm to determine the optimal set of coefficients $\{a_J\}$ that minimizes the spread of the wavepacket. Here, we use the basin hopping algorithm[9] to determine an optimal set $\{a_J\}$ that satisfies the above condition. To streamline the computations, we note that the number of polariton states within a given energy window is significantly smaller than the total number of states in the system. Thus, we use an iterative eigenvalue algorithm, specifically the ARPACK library,[10] to isolate the relevant polariton states for the optimization task.

**Nuclear Initial Conditions for the model systems**.

For the model used in Figs. 1-2 in the main text, initial bath conditions were sampled from the

analytic form of the Wigner distribution for a Gaussian bath

$$[\hat{\rho}_{\mathrm{R}}]_{\mathrm{W}}(\mathbf{R},\mathbf{P}) = \prod_{n,\nu} 2\tanh\left(\frac{\beta\hbar\omega_\nu}{2}\right)\exp\left\{-\tanh\left(\frac{\beta\hbar\omega_\nu}{2}\right)\left(\frac{\omega_\nu^2 R_{n,\nu}^2}{\hbar^2} + \frac{P_{n,\nu}^2}{\hbar^2\omega_\nu^2}\right)\right\} \tag{S40}$$

which are sampled using the Gaussian random number generator based on the Box–Muller method.

**$\mathcal{L}$-MFE Simulation Details**.

All results are obtained with an ensemble of 250 independent trajectories. Convergence tests are performed with up to 1000 trajectories. The nuclear time step used in $\mathcal{L}$-MFE method is $\Delta t = 2.5$ fs, where during each nuclear propagation, there are 100 electronic propagation steps with a time step $dt = 0.025$ fs. The nuclear EOM in Eq. S27 is numerically integrated with the velocity Verlet algorithm (with Force expression in Eq. S27) and the TDSE (Eq. S26) is solved with the RK4 algorithm. For each electronic time step, the Stochastic Lindblad loss described in Eq. S30-Eq. S31 is applied to update the photonic coefficients in order to capture the Lindblad loss dynamics of the cavity modes.

For the $\mathcal{L}$-MFE simulations, we sample Eq. S11 by using a total of $N_\nu = 35$ bath modes (for each molecule) for various values of $\lambda$ meV, and $\omega_b = 6.2$ meV. The bath parameters are sampled using the procedure outlined in Ref. 11 as follows

$$C_{n,\nu} = 2\sqrt{\lambda\frac{\tan^{-1}(\omega_{\max}\tau_\nu)}{\pi N_\nu \omega_\nu}}, \tag{S41a}$$

$$\omega_\nu = \frac{1}{\tau_\nu}\tan\left(\frac{\nu}{N_\nu}\tan^{-1}(\omega_{\max}\tau_\nu)\right), \tag{S41b}$$

where $\tau_\nu = 1/\omega_{\mathrm{b}}$, $\omega_{\max} \gg \omega_{\mathrm{b}}$ is the maximum frequency when discretizing the bath frequencies. Here, we choose $\omega_{\max} = 20\omega_{\mathrm{b}}$.

## IV. Details of the ab initio electronic structure and polariton transmission spectra simulations

For the transmission spectra shown in Fig. 3a,b in the main text, we consider a single independent-mode Hamiltonian as follows

$$\hat{H}_{\mathrm{pl}}(\mathbf{R}, k_\alpha) = \sum_{n=0}^{N-1} \hat{H}_{\mathrm{el}}(\mathbf{R}_n) + \omega_{k_\alpha} \hat{a}^\dagger_{k_\alpha} \hat{a}_{k_\alpha} + \sqrt{\frac{\omega_{k_\alpha}}{2}} \boldsymbol{\lambda}_{k_\alpha} \cdot \hat{\boldsymbol{\mu}}(\mathbf{R}_n)(\hat{a}^\dagger_{k_\alpha} + \hat{a}_{k_\alpha}), \tag{S42}$$

where $\hat{H}_{\mathrm{el}}(\mathbf{R}_n) \equiv \hat{H}_n - \hat{T}_{\mathbf{R}_n}$ is the electronic Hamiltonian of molecule $n$ [c.f. Eq. 1 of the main text] and $\boldsymbol{\lambda}_{k_\alpha} \equiv \sqrt{\frac{1}{\epsilon \mathcal{V}}} \hat{\boldsymbol{e}}_{\mathrm{TE}}$.

Solving the polaritonic eigenvalue equation (Eq. S42)

$$\hat{H}_{\mathrm{pl}}(\mathbf{R}, k_\alpha) \left| \Phi_{J,k_\alpha}(\mathbf{R}) \right\rangle = \mathcal{E}_{J,k_\alpha}(\mathbf{R}) \left| \Phi_{J,k_\alpha}(\mathbf{R}) \right\rangle, \tag{S43}$$

we obtain in mode-specific polaritonic eigenfunctions $|\Phi_{J,k_\alpha}(\mathbf{R})\rangle$ and eigenenergies $\mathcal{E}_{J,k_{\alpha}(\mathbf{R})}$. Here, we use the single excitation subspace, as described in Eq. 4 in the main text.

These eigenenergies and eigenstates are then used to compute the mode-specific $k_\alpha$-resolved (angle-resolved) transmission spectra through the following expression

$$\mathcal{T}_J(\omega, k_\alpha) = \left\langle \mathcal{N}_{J,k_\alpha} \cdot \delta(\hbar\omega - \mathcal{E}_{J,k_\alpha}(\mathbf{R})) \right\rangle_{\mathbf{R}}. \tag{S44}$$

Here, $\mathcal{N}_{J,k_\alpha} = \langle \Phi_{J,k_\alpha}(\mathbf{R}) | \hat{a}^\dagger_{\mathbf{k}} \hat{a}_{\mathbf{k}} | \Phi_{J,k_\alpha}(\mathbf{R}) \rangle$ is the photon number expectation value associated with polariton state $J$. The polaritonic density of states (delta function) was weighted by the photonic character for that polariton polariton branch to give the transmission spectra. The delta function in Eq. S44 was broadened with a Gaussian of width $\sigma \approx k_{\mathrm{B}}T = 26$ meV (room temperature fluctuation). The brightest two correspond to the UP and LP branches at a given $k_\alpha$ away from the zero light-matter detuning region (anti-crossing region).

The ensemble average $\langle ... \rangle_{\mathbf{R}}$ represents an average over geometries sampled from the Born-Oppenheimer Molecular Dynamics simulations, with a similar computational protocol reported in our earlier work.[12] In this work, we simulated 2 ps of Born-Oppenheimer molecular dynamics for 540

BODIPY molecules at $\Delta t = 1$ fs nuclear time step in the canonical ensemble (*i.e.*, constant NVT, with T = 300 K) using Langevin dynamics, as implemented in the SQD code[13] which interfaces with the Gaussian 16 software package.[14] The molecular ground state energies and forces were computed using the semi-empirical AM1 Hamiltonian[15] while the excited states' energy and dipoles of these organic molecules were computed using linear-response formalism in the Tamm-Dancoff approximation (TDA-AM1). The dipole-filed coupling term $\hat{\boldsymbol{\mu}}(\mathbf{R}_n)\cdot\hat{\mathbf{e}}_{\mathrm{TE}}$ is then computed, using the output of $\hat{\boldsymbol{\mu}}(\mathbf{R}_n)$ in Cartesian coordinates in 3D. Discarding the first 1.0 ps for equilibration, each of the 540 BODIPY molecules contains a 1000 fs-long trajectory, including data for the electronic transition energy and electronic transition electric dipole vectors. Note that we explicitly corrected the phase of the transition dipole, which is necessary due to the arbitrary phase of the transition density as obtained by the TDA eigenvalue equation.

To compute the mode-resolved TM spectra from the linear response frequency domain (Eq. S44), the Hamiltonian for each $k_\alpha$ (see Eq. S42 and Eq. S43) was built with 108 molecules, and the ensemble average was performed over a total of 5000 snapshots. Note that when computing by using Eq. S42, we are assuming the single-mode limit of the original many-mode Hamiltonian (Eq. S6). It has been theoretically justified that as long as a homogeneous molecular distribution is satisfied, the eigenspectrum and linear spectra would be identical, see Eq. 10-Eq. 16 in Ref. 16.

## V. Details of the ab initio polariton quantum dynamics simulations

**Parameters of Ab Initio CPA Simulations.** For the ab initio CPA simulations presented in Figs. 3-4 of the main text, we use $\tilde{N} = 108$ BODIPY molecules and $\mathcal{M} = 51$ cavity modes with the mode frequency obtained based on Eq. S4 The inter-molecular spacing is set to be $\tilde{L} = 1500$ Å, such that the simulation box size $\tilde{N}\tilde{L}$ is the same as for the model system. In simulations of the GHTC model Hamiltonian, the mean exciton energy from the ab initio simulation fluctuate around $E_e - E_g \approx 3.25$ eV, and the fundamental photon frequency was taken to be $\hbar\omega_{\mathrm{c}} \approx 3$ eV (which is $\omega_{\mathbf{k}}$ at the normal incidence $k_\parallel = 0$) for the transport results presented in Fig. 3C and Fig. 4 in the main text. For the transmission spectra presented in Fig. 3A-B in the main text, a different value of the cavity frequency $\hbar\omega_{\mathrm{c}} \approx 2.5$ eV was chosen, in order to clearly demonstrate the anti-crossing behavior under the strong coupling conditions as shown in Fig. 3b of the main text.

To connect with the experiments, one can estimate the inter-molecular distance of BODIPY molecules, based on the concentration as $L = 40$Å, based on the molecular concentration reported in Ref. 17. Here, for the ab initio simulation, we choose a much larger distance $\tilde{L} = sL$, where $s = 37.5$ is the scaling factor. Here, we demonstrate that as long as $\tilde{N}\tilde{L} = NL$, where $\tilde{N} = N/s$, the transport physics is preserved, given that $\Omega_\mathrm{R} \sim 2\sqrt{\tilde{N}}\tilde{\lambda}_\mathrm{c}$ is fixed. *i.e.* when choosing $\tilde{\lambda}_\mathrm{c} = \sqrt{s}\lambda_\mathrm{c}$.

This is because for the original system with intersite distance $L$, $k_\alpha = \frac{2\pi}{NL} \cdot \alpha$, with $\alpha \in [-N/2, N/2]$. With a scaled parameter choice,

$$\begin{aligned}\tilde{k}_\alpha &= \frac{2\pi}{\tilde{N}\tilde{L}} \cdot \alpha \ \text{ with } \ \alpha \in \left[-\frac{\tilde{N}}{2}, ..., \frac{\tilde{N}}{2}\right] \\ &= \frac{2\pi}{\frac{N}{s} \cdot sL}\alpha \ \text{ with } \ \alpha \in \left[-\frac{N}{2s}, ..., \frac{N}{2s}\right] \\ &= \frac{2\pi}{NL}\alpha \ \text{ with } \ \alpha \in \frac{1}{s}\left[-\frac{N}{2}, ..., \frac{N}{2}\right]\end{aligned}$$

As such, by scaling $L$ to $\tilde{L} = sL$, we are effectively sampling $\frac{1}{s}$-th fraction with the same Brillouin zone with $\frac{1}{s}$-th number of grid points as earlier. On the other hand, the $k$-grid point density does not change within the Brillouin zone $k_\alpha \in \left[-\frac{N}{2s}, ..., \frac{N}{2s}\right]$, becaue the grid point distance $\Delta k = \frac{2\pi}{\tilde{N}\tilde{L}} = \frac{2\pi}{NL}$. The only thing that is changing is the part of the Brillouin Zone (BZ) that is being considered in the simulation. By choosing $s = 37.5$, we are considering $1/s \approx 3\%$ of the full 1st BZ, which covers the range of disorerson we reported in Fig. 3b. As such, this choice captures the important part of the transport dynamics within the relevant Brillouin Zone. Further, this will not impact the light-matter coupling term in Eq. 2 of the main text. Recall that the phase factor in the coupling term is

$$e^{-ik_\alpha x_n} = \exp\left[-i\frac{2\pi}{NL} \cdot \alpha \cdot (Ln)\right] = \exp\left[-i\frac{2\pi}{\tilde{N}\tilde{L}} \cdot \alpha \cdot (\tilde{L} \cdot \frac{n}{s})\right] \tag{S45}$$

where we have fewer sites, but the phase factor remains the same. This gives fewer terms in the sum of the number of molecules $\tilde{N}$ On the other hand, our choice of $\tilde{N}$ gives a much smaller number of dark states, which is $\tilde{N} - \mathcal{M}$ that is on the same order as the number of polariton states $\mathcal{M}$. For the result presented in this work, which has an initial UP excitation and decay to dark, the transport dynamics are not significantly impacted.

**Initial Polariton Condition**. For the ab initio CPA simulations to compute the MSD (Fig. 3c

and Fig. 4 in the main text), the polariton initial condition was prepared on UP state, with the same numerical methods described in Eq. S36 to Eq. S39, with a center energy of UP as $\mathcal{E}_0 = 3.38$ eV, and a energy window $\Delta\mathcal{E} = 0.1$ eV.

For the classical path approximation (CPA), the nuclear trajectories for molecule $n$, $\mathbf{R}_n(t)$, are pre-computed using the ground state forces

$$\mathbf{F}_n = -\nabla_n E_g(\mathbf{R}_n(t)). \tag{S46}$$

These trajectories are the same data as those used for the generation of the mode-resolved transmission spectra outlined in the previous Section.

We expand exciton-photonic degrees of freedom (DOF) quantum mechanically

$$|\psi(t)\rangle = \sum_{n=1}^{N} c_n(t)\,|E_n(\mathbf{R}(t))\rangle + \sum_{\alpha} c_\alpha(t)\,|k_\alpha\rangle\,. \tag{S47}$$

The polariton quantum dynamics is propagated with

$$i\hbar\frac{\partial}{\partial t}|\psi(t)\rangle = \hat{H}_{\mathrm{Q}}(\mathbf{R}(t))|\psi(t)\rangle, \tag{S48}$$

where $|\psi(t)\rangle$ is represented using Eq. S47, $\hat{H}_{\mathrm{Q}} = \hat{H} - \sum_n \hat{T}_{\mathbf{R}_n}$ is the polariton quantum subsystem Hamiltonian including excitonic and photonic DOFs without the nuclear kinetic energy [c.f. Eq. 2 and Eq. 6 of the main text]. We use $N = 108$ molecules coupled to the cavity in order to perform the *ab initio* CPA simulation, allowing us to have five independent realizations (*ab initio* CPA trajectories) for solving Eq. S48. The details of the electronic structure are provided in the previous section. We use $\mathcal{L}$-MFE dynamics approach[8,18,19] to simulate the polariton transport quantum dynamics in a lossy cavity for the *ab initio* systems, through stochastic Lindblad dynamics using the same algorithm described in Eq. S30-Eq. S33. The EOM in Eq. S48 is numerically solved using the RK4 algorithm, with a time step $dt = 1/500$ fs.

**Spacial Distribution of the Polariton Wavepacket**. To obtain the spatial distribution of the polariton wavepacket at time $t$ and position $x_n$ for *ab initio* transport simulations, we

diagonalize $\hat{H}_\mathrm{Q}(\mathbf{R}(t))$ to obtain

$$\hat{H}_\mathrm{Q}(\mathbf{R}(t))\,|\Phi_{J,\xi}(\mathbf{R}(t))\rangle = \mathcal{E}_{J,\xi}(\mathbf{R}(t))\,|\Phi_{J,\xi}(\mathbf{R}(t))\rangle\,, \tag{S49}$$

where $|\Phi_{J,\xi}(\mathbf{R}(t))\rangle$ and $\mathcal{E}_{J,\xi}(\mathbf{R}(t))$ are the eigenvectors and corresponding eigenenergies of the Hamiltonian. Here, we use the label $\xi$ to denote which polaritonic state the eigenvectors correspond to, that is $\xi \in \{+, -, \mathrm{D}\}$, and the index $J$ represents the inclusion of states in that particular polaritonic class. The spatial distribution of the polariton wavepacket is given by the projection onto the polaritonic eigenvectors, such that for the UP and LP wavepackets, we have

$$\psi_\pm(x_n,t) = \sum_J \langle E_n|\Phi_{J,\pm}(\mathbf{R}(t))\rangle\langle\Phi_{J,\pm}(\mathbf{R}(t))|\psi(t)\rangle, \tag{S50}$$

and for the dark states wavepacket, we have

$$\psi_\mathrm{D}(x_n,t) = \sum_J \langle E_n|\Phi_{J,\mathrm{D}}(\mathbf{R}(t))\rangle\langle\Phi_{J,\mathrm{D}}(\mathbf{R}(t))|\psi(t)\rangle, \tag{S51}$$

The contribution from UP and dark state are expressed as

$$|\psi(x_n,t)|^2 = |\psi_+(x_n,t)|^2 + |\psi_\mathrm{D}(x_n,t)|^2. \tag{S52}$$

The transient MSD is expressed as

$$\sigma^2(t) = \langle\psi(t)|(\hat{x} - \langle\hat{x}\rangle)^2|\psi(t)\rangle = \sum_n |\psi(x_n,t)|^2 \cdot (x_n - \langle\hat{x}\rangle)^2, \tag{S53}$$

where $\langle\hat{x}\rangle$ is the centroid of the initial polariton wavepacket (at $t = 0$) in position space. We use this expression to compute the transient MSD for the ab initio CPA simulation reported in Fig. 3c and 4 of the main text.

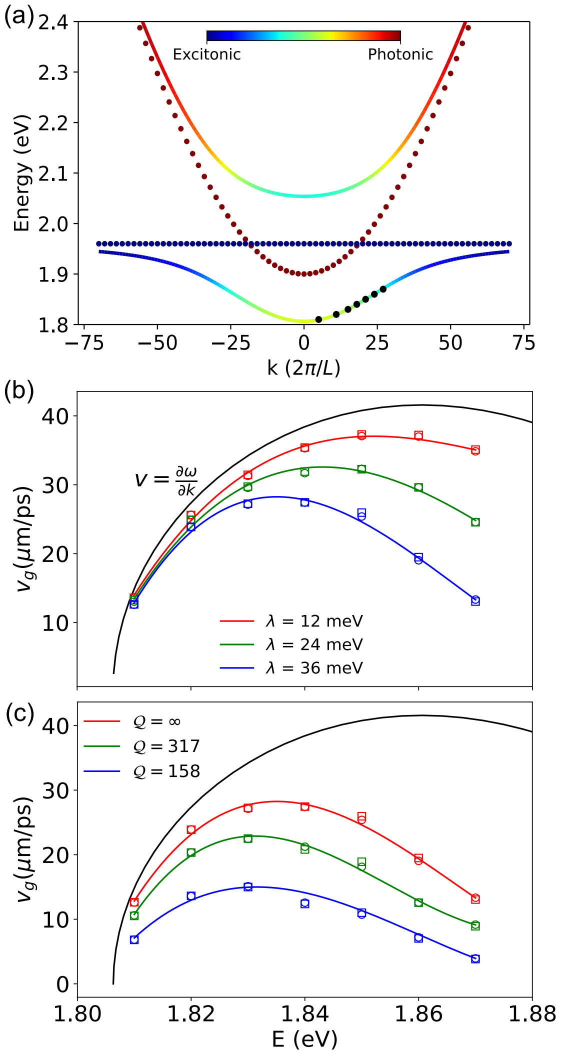


Figure S2: (a) Dispersion curve of the photon (red dots) and matter (blue dots) (b) Group velocities $v_g$ of polaritons for various reorganization energy $\lambda$ in a lossless cavity. (c) Group velocities $v_g$ of polaritons for different cavity quality factor $\mathcal{Q}$ with phonon reorganization energy $\lambda = 36$ meV. The open circles in (b) and (c) are evaluated from wavepackets with quantum forces on the nuclei, and the open squares are evaluated from wavepackets with the CPA.

## VI. Additional Numerical Results

### Polariton Group Velocity Calculation with CPA using model systems

Fig. S2a presents the energy diagrams for the UP and LP bands formed from hybridizing the photonic band and the excitonic band. The LP and UP bands are color-coded based on their photonic character. The collective light-matter coupling strength is $\sqrt{N}g_{\rm c} = 120$ meV. These polariton states

are analytically expressed in Eq. S16 . The initial excitation conditions are indicated in Fig. S2 using black dots on the LP branch, which corresponds to a pulse with a narrow energy bandwidth (to model the experimental condition in Ref. 3). The group velocity is extracted using the algorithm detailed in the previous work,[3,5] *e.g.*, see Eq. S60-Eq. S61 in the Supporting Information of Ref. 5.

Fig. S2b and S2c shows $v_g$ with different initial energies (corresponding to different $k_{\|}$ in Fig. S2a). In Fig. S2b, we show results for a lossless cavity ($\Gamma_c = 0$) with varying reorganization energy, and in Fig. S2c, we show cavities with varying $\mathcal{Q}$ factor while fixing the bath parameters ($\lambda$ and $\omega_f$). The solid black line indicates the group velocity obtained as $v_g = \partial\omega_-/\partial k_{\|}$. The open circles with different colors are $v_g$ for the excitonic system with different reorganization energy $\lambda$ in Fig. S2b, and different $\mathcal{Q}$ factor in Fig. S2c. With an increase in $\lambda$, $v_g$ decreases, which indicates that the polaritons move at a reduced $v_g$ due to increased exciton-phonon coupling, which was previously referred to as the group velocity renormalization.[3] Similarly, decreasing $\mathcal{Q}$ causes $v_g$ to decrease due to rapid attenuation of the photonic contribution to the polariton wavepacket. Increasing the excitonic character (increasing $E$) causes more renormalization of $v_g$, in agreement with the results in Ref. 3. The open squares with different colors are $v_g$ computed with the CPA. We see from these results that $v_g$ computed from wavepackets propagated using the CPA is in good agreement with $v_g$ computed with wavepackets propagated by evaluating all contributions from the nuclear force terms.

## VII. Further Discussions on CPA

The classical-path approximation (CPA),[20–22] drastically reduces the cost of the electronic structure calculations and enables large-scale, accurate quantum dynamics simulations. However, when having a small number of quantum states, CPA often faces challenges in providing accurate quantum dynamics for ab initio simulation of photochemistry[20,23] and in spin-boson model dynamics (especially at a low temperature[24,25]), due to the non-negligible nuclear forces. In this work, we theoretically demonstrate that CPA remains valid for the polariton transport dynamics, as long as the dynamics themselves are delocalized among many excitonic states. To the best of our knowledge, this is the first time that the general validity of CPA in polariton transport dynamics is theoreti-

cally investigated, as well as being used in combination with the ab initio on-the-fly simulations to investigate the transport dynamics.

Note that Ref. 26 assumes CPA and derived an analytic expression for group velocity renormalization, which agrees with the MFE simulations that do not assume CPA. This previous work provides an indirect test of the validity of CPA, and focuses only on group velocity, which does not contain the full information of transport. Here, we provide a direct test of CPA by performing the same level of simulations with the full nuclear gradient and with CPA, to directly test every possible quantum dynamical behavior of transport, including MSD, group velocity, population dynamics, and spatial-temporal polariton wavepacket. Because of these explicit and direct tests of CPA, we realized the reason behind the validity of CPA, which is that the delocalization of the wavepacket makes the approximation valid.

Theoretically, it is also possible to create a local initial excitation at just one site. We expect that the validity of CPA will break down at least for the early time dynamics when polaritons are so localized among just a few molecules. On the other hand, as the dynamics progress, the wavepacket will spread out in real space, due to the collective light-matter couplings where cavity modes are coupled to all molecular excitations (c.f. Eq. S1), and we expect that CPA will regain validity for those delocalized stages of the dynamics.

For a higher excitation subspace, such that exciton-exciton interactions become important (in the context of many-body quantum dynamics of exciton polaritons), CPA could have a higher chance to break down.[27] This is because[27] the non-adiabatic, state-dependent force $\propto N_{\mathrm{ex}} \cdot |c_n(t)|^2 \nabla_n [E_e(\mathbf{R}_n) - E_g(\mathbf{R}_n)]$, where $N_{\mathrm{ex}}$ is the number of excitons in the system,[27] which itself could be large. in Ref. 27, up to $N_{\mathrm{ex}} \sim 10^3$ was investigated. On the other hand, as long as the dynamics are delocalized, $|c_n(t)|^2 \sim 1/\mathcal{N}$, so as long as $N_{\mathrm{ex}} \ll \mathcal{N}$ in the simulation (say $\mathcal{N} \propto N \sim 10^4 - 10^6$), CPA should remain valid even for the case of transport in the higher excitation subspace. Of course, for ab initio simulations, this means that more molecules are needed when considering a large $N_{\mathrm{ex}}$. Finally, our work is limited by the accuracy of the MFE dynamics, which itself could be less accurate for non-adiabatic dynamics compared to the other available trajectory-based method.[19] The validity of CPA in MFE simulations suggests that the state-dependent nuclear forces play a negligible role in the polariton transport dynamics. Nevertheless, the CPA ground

state forces are still based on a classical approximation, and one could replace them with ground state quantum forces for the nuclei in future investigations.

# References


(1) Mandal, A.; Taylor, M. A.; Weight, B. M.; Koessler, E. R.; Li, X.; Huo, P. Theoretical advances in polariton chemistry and molecular cavity quantum electrodynamics. *Chemical Reviews* **2023**, *123*, 9786–9879.

(2) Tichauer, R. H.; Feist, J.; Groenhof, G. Multi-scale dynamics simulations of molecular polaritons: The effect of multiple cavity modes on polariton relaxation. *The Journal of Chemical Physics* **2021**, *154*, 104112.

(3) Xu, D.; Mandal, A.; Baxter, J. M.; Cheng, S.-W.; Lee, I.; Su, H.; Liu, S.; Reichman, D. R.; Delor, M. Ultrafast imaging of polariton propagation and interactions. *Nature Communications* **2023**, *14*, 3881.

(4) Tichauer, R. H.; Sokolovskii, I.; Groenhof, G. Tuning the Coherent Propagation of Organic Exciton-Polaritons through the Cavity Q-factor. *Advanced Science* **2023**, *10*, 2302650.

(5) Chng, B. X.; Mondal, M. E.; Ying, W.; Huo, P. Quantum Dynamics Simulations of Exciton Polariton Transport. *Nano Letters* **2025**, *25*, 1617–1622.

(6) Nitzan, A. *Chemical dynamics in condensed phases: relaxation, transfer and reactions in condensed molecular systems*; Oxford university press, 2006.

(7) Qiu, L.; Mandal, A.; Morshed, O.; Meidenbauer, M. T.; Girten, W.; Huo, P.; Vamivakas, A. N.; Krauss, T. D. Molecular polaritons generated from strong coupling between CdSe nanoplatelets and a dielectric optical cavity. *The Journal of Physical Chemistry Letters* **2021**, *12*, 5030–5038.

(8) Koessler, E. R.; Mandal, A.; Huo, P. Incorporating Lindblad decay dynamics into mixed quantum-classical simulations. *The Journal of Chemical Physics* **2022**, *157*, 064101.

(9) Wales, D. J.; Doye, J. P. Global optimization by basin-hopping and the lowest energy structures of Lennard-Jones clusters containing up to 110 atoms. *The Journal of Physical Chemistry A* **1997**, *101*, 5111–5116.

(10) Lehoucq, R. B.; Sorensen, D. C.; Yang, C. *ARPACK users' guide: solution of large-scale eigenvalue problems with implicitly restarted Arnoldi methods*; SIAM, 1998.

(11) Huo, P.; Coker, D. F. Semi-classical path integral non-adiabatic dynamics: a partial linearized classical mapping Hamiltonian approach. *Mol. Phys.* **2012**, *110*, 1035–1052.

(12) Weight, B.; Rury, A.; Shao, Y.; Huo, P. Ab Initio Polariton Spectra of ZnTPP Molecules Collectively Coupled inside an Optical Cavity. *ChemRxiv* **2025**, 10.26434/chemrxiv–2025–r98bz.

(13) Weight, B. M.; Mandal, A.; Huo, P. Semiclassical Quantum Dynamics (SQD). 2023; `https://github.com/bradenmweight/SQD`, original-date: 2023-01-15T22:28:05Z.

(14) Frisch, M. J. et al. Gaussian˜16 Revision C.01. 2016; Gaussian Inc. Wallingford CT.

(15) Dewar, M. J. S.; Zoebisch, E. G.; Healy, E. F.; Stewart, J. J. P. Development and use of quantum mechanical molecular models. 76. AM1: a new general purpose quantum mechanical molecular model. *Journal of the American Chemical Society* **1985**, *107*, 3902–3909.

(16) Li, T. E. Vibrational polaritons with broken in-plane translational symmetry. *The Journal of Chemical Physics* **2024**, *161*, 064308.

(17) Pandya, R.; Ashoka, A.; Georgiou, K.; Sung, J.; Jayaprakash, R.; Renken, S.; Gai, L.; Shen, Z.; Rao, A.; Musser, A. J. Tuning the coherent propagation of organic exciton-polaritons through dark state delocalization. *Advanced Science* **2022**, *9*, 2105569.

(18) Mondal, M. E.; Koessler, E. R.; Provazza, J.; Vamivakas, A. N.; Cundiff, S. T.; Krauss, T. D.; Huo, P. Quantum dynamics simulations of the 2D spectroscopy for exciton polaritons. *The Journal of Chemical Physics* **2023**, *159*, 094102.

(19) Hu, D.; Chng, B. X. K.; Ying, W.; Huo, P. Trajectory-based non-adiabatic simulations of the polariton relaxation dynamics. *J. Chem. Phys.* **2025**, *162*, 124113.

(20) Akimov, A. V.; Prezhdo, O. V. The PYXAID program for non-adiabatic molecular dynamics in condensed matter systems. *Journal of chemical theory and computation* **2013**, *9*, 4959–4972.

(21) Wang, L.; Akimov, A.; Prezhdo, O. V. Recent progress in surface hopping: 2011–2015. *The journal of physical chemistry letters* **2016**, *7*, 2100–2112.

(22) Kang, J.; Wang, L.-W. Nonadiabatic molecular dynamics with decoherence and detailed balance under a density matrix ensemble formalism. *Physical Review B* **2019**, *99*, 224303.

(23) Nijjar, P.; Jankowska, J.; Prezhdo, O. V. Ehrenfest and classical path dynamics with decoherence and detailed balance. *The Journal of Chemical Physics* **2019**, *150*, 204124.

(24) Makri, N. Exploiting classical decoherence in dissipative quantum dynamics: Memory, phonon emission, and the blip sum. *Chemical Physics Letters* **2014**, *593*, 93–103.

(25) Wang, F.; Makri, N. Quantum-classical path integral with a harmonic treatment of the back-reaction. *The Journal of Chemical Physics* **2019**, *150*, 184102.

(26) Blackham, L.; Manjalingal, A.; Rahmanian Koshkaki, S.; Mandal, A. Microscopic Theory of Polaron-Polariton Dispersion and Propagation. *Nano Letters* **2025**, *25*, 15874–15882.

(27) Ghosh, P.; Manjalingal, A.; Wickramasinghe, S.; Rahmanian Koshkaki, S.; Mandal, A. Mean-field mixed quantum-classical approach for many-body quantum dynamics of exciton polaritons. *Physical Review B* **2025**, *112*, 104319.
